# Mapping metabolic reprogramming in lung and breast cancer through integrative bioinformatics

**DOI:** 10.1371/journal.pone.0350628

**Published:** 2026-06-04

**Authors:** Nosayba Al-Damook, Molham Sakkal, Mostafa Khair, Walaa K. Mousa, Rose Ghemrawi

**Affiliations:** 1 College of Pharmacy, Al Ain University, Abu Dhabi, United Arab Emirates; 2 AAU Health and Biomedical Research Center, Al Ain University, Abu Dhabi, United Arab Emirates; 3 Core Technology Platforms, New York University Abu Dhabi, Abu Dhabi, United Arab Emirates; 4 College of Pharmacy, Mansoura University, Mansoura, Egypt; Apeejay Stya University, INDIA

## Abstract

Metabolic reprogramming is central to cancer biology, enabling tumor cells to sustain rapid proliferation, resist stress, and adapt to therapy. However, these alterations are highly heterogeneous across cancer types, and current treatments rarely exploit subtype-specific metabolic vulnerabilities. To address this gap, we developed a unified bioinformatics framework that integrates transcriptomic profiling (UALCAN), drug–gene interactions (DGIdb), gene–disease associations (Open Targets), pathway enrichment (Enrichr), and protein–protein interaction networks (STRING/Cytoscape). This pipeline was applied to lung adenocarcinoma (LUAD), lung squamous cell carcinoma (LSCC), breast cancer (BRCA), and metastatic breast tumors (MET500) to uncover cancer type–specific metabolic programs and prioritize translational targets. Our analysis revealed distinct signatures: LUAD showed glycolytic activation, LSCC coupled glycolysis with oxidative phosphorylation, BRCA favored anabolic and lipogenic pathways, and MET500 tumors adopted stress-adaptive states with elevated antioxidant and autophagy programs. Integration of pharmacological evidence highlighted clinically actionable interactions between metabolic genes and FDA-approved drugs, including ASNS–asparaginase, DHODH–teriflunomide, and G6PD–rasburicase. Gene–disease associations further prioritized G6PD, SLC2A1, and TK1 as robust targets strongly linked to lung and breast cancers. Pathway enrichment pinpointed the pentose phosphate pathway, pyrimidine metabolism, and glutathione metabolism as conserved axes sustaining tumor survival, while network analysis positioned the G6PD–PGD hub as a central metabolic node connecting glucose uptake, redox balance, and nucleotide biosynthesis. To place these bioinformatics-derived findings within a functional and clinical context, we complemented the computational analyses with patient survival assessment, clinical trial screening, and targeted literature appraisal. Survival analysis demonstrated cancer type–specific prognostic relevance for selected metabolic genes, while clinical and literature-based screening revealed both ongoing translational efforts and substantial gaps between computational target prioritization and experimental or clinical validation. This integrative analysis shows that cancer metabolism is altered in subtype-specific ways that can be systematically mapped to reveal potential therapeutic targets. By linking transcriptomic evidence with drug–gene interactions and clinical context, this framework provides a scalable approach for cancer metabolism research and supports the prioritization of pathways with potential translational relevance.

## Introduction

Cancer cells survive and proliferate by fundamentally reshaping their metabolism [1,2]. They increase glucose uptake and favor glycolysis even in oxygen-rich conditions, accelerate lipid and nucleotide biosynthesis, reroute amino acid metabolism, and enhance antioxidant defenses [3]. These metabolic alterations supply the energy and building blocks necessary for rapid growth while enabling resistance to stress and therapy [4]. Importantly, such reprogramming is not uniform across cancers. For example, it was found that Epithelial–mesenchymal transition (EMT) drives cancer progression through cancer type–specific metabolic reprogramming, even within the same organ, as demonstrated by transcriptome profiling across 31 cancers including lung adenocarcinoma (LUAD), lung squamous cell carcinoma (LSCC), breast cancer (BRCA), stomach cancer (STAD), and others, which revealed distinct metabolic signatures associated with immune microenvironments, patient prognosis, and potential therapeutic targets [5,6]. Despite this heterogeneity, current clinical practice rarely accounts for subtype-specific metabolic vulnerabilities, and patients with fundamentally different metabolic profiles are often treated with similar regimens [7]. This disconnect highlights an urgent need for systematic strategies that can map metabolic dependencies across cancers and link them to clinically actionable therapeutic opportunities.

The rapid expansion of bioinformatics resources has created an unprecedented opportunity to study cancer metabolism at scale. Datasets such as UALCAN [8] provide expression profiles across cancers, DGIdb [9] curates drug–gene interactions, Open Targets integrates gene–disease associations [10], Enrichr [11] enables pathway and functional enrichment analysis, and STRING [12] offers protein–protein interaction networks. While each of these platforms generates valuable insights, they are often applied in isolation, producing fragmented perspectives that are difficult to translate into clinical applications.. What remains needed is an integrative framework that not only unifies expression, pharmacological, and functional evidence, but also places bioinformatics-derived findings within a clinically meaningful context.

**Fig 1:**
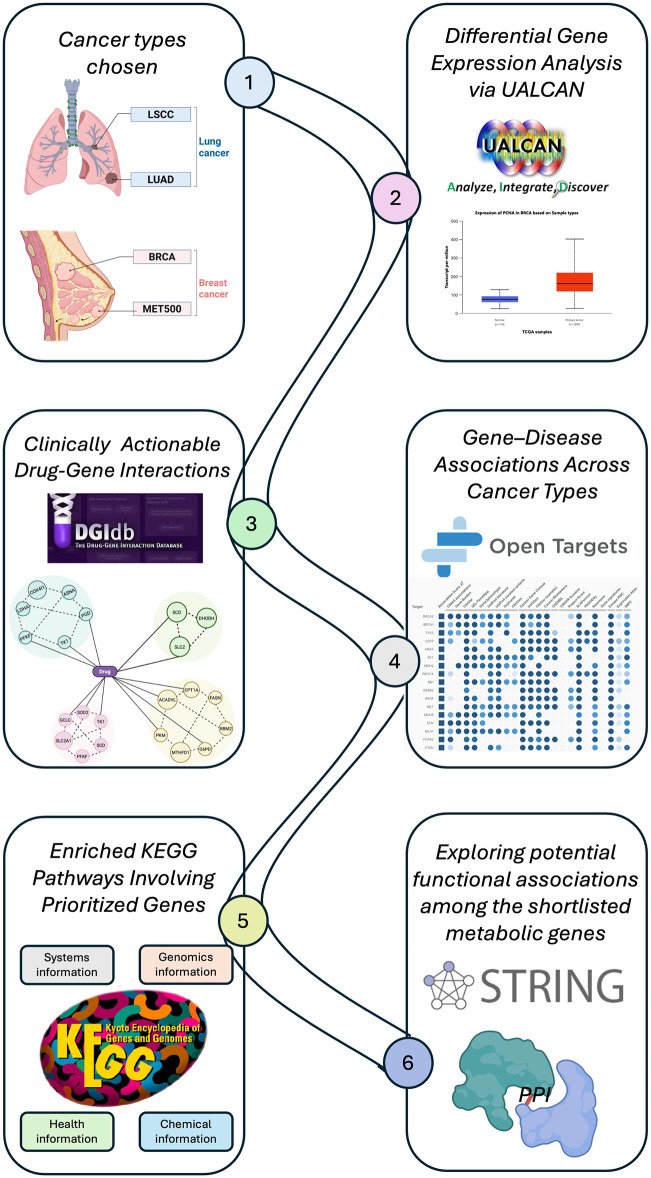
Integrative bioinformatics pipeline for mapping cancer type–specific metabolic vulnerabilities. The workflow illustrates integration of multiple resources to characterize metabolic reprogramming across cancers. (1) Cancer types analyzed included lung adenocarcinoma (LUAD), lung squamous cell carcinoma (LSCC), breast cancer (BRCA), and metastatic breast tumors (MET500). (2) Differential gene expression analysis was performed using UALCAN. (3) Clinically actionable drug–gene interactions were identified through DGIdb. (4) Gene–disease associations across cancer types were explored via Open Targets. (5) Prioritized genes were subjected to pathway enrichment using Enrichr/KEGG databases. (6) Protein–protein interaction (PPI) networks were constructed with STRING/Cytoscape to examine functional associations among shortlisted metabolic genes.

In this study, we implemented an integrative bioinformatics pipeline (Fig 1) by systematically combining multiple established platforms to prioritize clinically relevant metabolic targets. Differentially expressed metabolic genes across LUAD, LSCC, BRCA, and metastatic breast tumors (MET500) were first identified using UALCAN. These candidates were then mapped to FDA-approved drug interactions through DGIdb to highlight clinically actionable targets. Their biological and translational relevance was further assessed using gene–disease association profiling from Open Targets. To place these genes within functional contexts, KEGG pathway and Gene Ontology enrichment analyses were performed using Enrichr. Finally, protein–protein interaction networks were generated through STRING and visualized in Cytoscape to reveal functional connectivity and central metabolic hubs. To further strengthen the biological and clinical relevance of this framework, we complemented the computational analyses with patient survival assessment, clinical trial screening of prioritized drug–gene interactions, and targeted literature appraisal of existing experimental evidence. This layered approach was designed to bridge computational discovery with clinical relevance, allowing us to evaluate not only which metabolic genes are altered, but also whether they are associated with patient outcomes, translational efforts, or experimental validation, and uncovers opportunities for therapeutic repurposing.

To our knowledge, this is the first study to integrate UALCAN, DGIdb, Open Targets, Enrichr, and STRING into a unified pipeline specifically designed for cancer metabolism research. By applying this framework, we uncovered strikingly distinct metabolic programs across cancers: LUAD displayed a glycolytic shift, LSCC an oxidative phenotype, BRCA elevated lipogenic and anabolic pathways, and MET500 tumors broad stress-adaptive and autophagy signatures. Importantly, we identified clinically actionable interactions such as ASNS–asparaginase, DHODH–teriflunomide, and G6PD–rasburicase, prioritized high-confidence targets including G6PD, SLC2A1, and TK1, and revealed central metabolic hubs such as the G6PD–PGD axis.

## Materials and methods

### Differential gene expression analysis via UALCAN

Differential expression analysis of metabolic genes was conducted using the UALCAN portal [8] (http://ualcan.path.uab.edu), which provides normalized RNA-seq expression data from The Cancer Genome Atlas (TCGA) and related datasets. Four cancer types were examined: LUAD, LSCC, BRCA, and MET500. TCGA datasets provided paired tumor and normal profiles for LUAD, LSCC, and BRCA, while MET500 offered expression data from metastatic lesions, enabling both primary–normal and primary–metastatic comparisons.

A panel of genes was selected to represent key metabolic and stress-related pathways relevant to cancer biology. These included glycolysis (SLC2A1, HK2, PFKFB3, LDHA, and PKM2), oxidative phosphorylation (PPARGC1A, NDUFS3, SDHA, and COX4I1), fatty acid metabolism (CPT1A, FASN, SCD1, and ACADVL), the pentose phosphate pathway (G6PD, TALDO1, and PGD), one-carbon metabolism (MTHFD2, SHMT1, and SHMT2), nucleotide biosynthesis (DHODH, RRM2, and TK1), amino acid metabolism (PHGDH, ASNS, and MTHFD1), redox balance and detoxification (NFE2L2, SOD2, GPX4, and GCLC), and autophagy and ER stress response (MAP1LC3B, ATG7, DDIT3, and HSPA5). For each of these genes, UALCAN-derived outputs were extracted and organized into a structured table that captured pathway classification, gene identity, biological function, and relevance in cancer. In addition, the dataset included mean expression levels in tumor versus normal samples, log₂-transformed fold change values, statistical significance (p-values), the direction of regulation (up- or downregulated in tumor samples), and the number of tumor and normal samples analyzed for each cancer type.

This comprehensive dataset provided a quantitative overview of gene expression alterations across LUAD, LSCC, BRCA, and MET500. The data were then used to generate heat maps constructed from log₂ fold-change values, which enabled the visualization of global expression patterns across pathways and cancer types.

### Identification of clinically relevant drug–gene interactions

To explore the therapeutic relevance of the analyzed genes, drug–gene interaction data were retrieved using the Drug–Gene Interaction Database (DGIdb), available at https://dgidb.org https://doi.org/10.1093/nar/gkad1040. DGIdb integrates information from multiple sources, including DrugBank, PharmGKB, and ChEMBL, and provides curated data on known or predicted interactions between genes and chemical compounds. The previously categorized gene sets identified through UALCAN were submitted to DGIdb using its online search interface. Retrieved interactions were filtered based on the following inclusion criteria: (1) FDA-approved drugs and (2) an interaction score equal to or greater than 2.0, [13,14]. This filtering prioritized clinically actionable targets in cancer metabolism and highlighted candidates for drug repurposing.

### Survival analysis using UALCAN

Overall survival analysis for the selected genes (ASNS, TK1, PGD, G6PD, SLC2A1, ACADVL, and DHODH) was conducted using the UALCAN portal (http://ualcan.path.uab.edu), which provides Kaplan–Meier survival plots derived from TCGA clinical datasets. Survival was evaluated for LUAD, LSCC, and BRCA. Because the MET500 dataset does not include patient survival metadata, no survival analysis was conducted for metastatic breast tumors.

For each gene and cancer type, the “Survival Analysis” module in UALCAN was used to compare overall survival between high-expression and low/medium-expression patient groups. UALCAN automatically assigns samples to these categories based on normalized transcript expression. Kaplan–Meier plots and corresponding log-rank test p-values were retrieved directly from the platform. All survival curves, p-values, and group stratifications reported in the study were generated by UALCAN’s built-in survival analysis tool.

### Identification of clinical trial evidence for DGIdb-identified drugs

To assess the clinical relevance of the DGIdb-identified drugs, each drug was systematically screened on ClinicalTrials.gov. Searches were performed using the drug name as the primary keyword under the “Intervention/Treatment” field. Because dataset abbreviations such as LUAD, LSCC, BRCA, and MET500 are not used as clinical condition labels, the corresponding clinically recognized terms were applied in the “Condition or Disease” field. Specifically, “Lung Adenocarcinoma” was used for LUAD, “Lung Squamous Cell Carcinoma” for LSCC, “Breast Cancer” for BRCA, and “Metastatic Breast Cancer” for MET500. Searches were restricted to interventional studies involving adult participants, and only Phase I–III trials across all recruitment statuses were included. For each drug, we documented whether any relevant clinical trials existed within these cancer types and extracted the associated NCT number(s) and recruitment status.

### Literature-based experimental validation of bioinformatics findings

To validate the findings generated by the applied bioinformatics tools and to assess potential gaps between computational predictions and experimental evidence, a targeted literature search was conducted using the Scopus and PubMed databases.

Search queries were limited to combinations of cancer types (LUAD, LSCC, BRCA, and MET500) and drug names identified through DGIdb analysis (including asparaginase, penciclovir, teriflunomide, thioctic acid, penicillamine, rasburicase, phenazopyridine, deoxycholic acid, and pegloticase).

The search was restricted to peer-reviewed research articles published in English within the last 10 years (2015–2025). Only studies reporting experimental work, including in vitro, in vivo, computational studies, or clinically relevant experimental investigations, were considered eligible. Review articles, conference abstracts, editorials, and non–English-language publications were excluded. Relevant studies were manually screened to confirm experimental validation within oncological settings.

### Gene–disease association profiling using open targets

Gene–disease association data for LUAD, LSCC, BRCA, and MET500 were manually collected from the Open Targets Platform (https://platform.opentargets.org/). For each gene, two key metrics were extracted: the Association Score, a normalized value (0–1) representing the overall strength of evidence linking the gene to the disease; and the Europe PMC score, which reflects literature-based evidence derived from text mining of scientific publications. This approach aided in prioritizing cancer-specific metabolic genes with strong supporting evidence [15].

### KEGG pathway enrichment analysis

KEGG pathway enrichment was performed using Enrichr (https://maayanlab.cloud/Enrichr/) with a set of cancer-related genes that showed consistent overexpression, clinically actionable drug interactions, and strong disease associations. The KEGG 2021 Human database was selected, and enriched pathways were ranked based on adjusted p-values and combined scores. Pathways with an adjusted p-value < 0.05 were considered significant [16].

### Gene ontology (GO) biological process enrichment

Gene Ontology (GO) enrichment analysis was performed using the Enrichr platform (https://maayanlab.cloud/Enrichr/enrich) to identify biological processes associated with the selected genes. The “GO Biological Process 2025” database was used to assess the functional roles of these genes. The gene list, selected based on prior analyses, was uploaded, and enrichment was evaluated using adjusted p-values, odds ratios, and combined scores. Only terms with statistically significant adjusted p-values were included in the results [17].

### Protein–protein interaction network analysis and visualization

Protein–protein interaction (PPI) analysis was performed to explore potential functional relationships among the shortlisted genes. The selected genes were submitted to the STRING database (version 12.0; https://string-db.org/) to retrieve high-confidence interaction data based on experimental evidence, co-expression, database annotations, and text mining. Interactions with a combined confidence score above 0.4 were retained [18].

The resulting interaction table was exported from STRING in TSV format and imported into Cytoscape (version 3.10.0) for network visualization. Nodes represented genes, and edges represented interactions weighted by the STRING combined score. Edge thickness was scaled using continuous mapping to reflect interaction strength, and node layout was adjusted for clarity [19].

## Results

### Identification of differentially expressed metabolic genes in cancer

Differential expression analysis of 33 metabolic genes was conducted using the UALCAN portal, which provides normalized RNA-seq data from TCGA and related datasets, and revealed distinct and cancer type–specific transcriptional programs across LUAD, LSCC, BRCA, and MET500. Within glycolysis, SLC2A1 was markedly upregulated in both LUAD and LSCC compared to normal lung tissue, consistent with its role as a glucose transporter, while LDHA and PKM2 were also significantly elevated, indicating reinforcement of aerobic glycolysis. In contrast, PFKFB3 showed significant downregulation in both LUAD and LSCC, whereas HK2 was modestly increased only in LSCC. Together, these results highlighted a glycolytic bias, most pronounced in LUAD.

In the oxidative phosphorylation pathway, LUAD and LSCC showed upregulation of mitochondrial subunits NDUFS3, SDHA, and COX4I1, whereas PPARGC1A (PGC-1α), a regulator of mitochondrial biogenesis, was significantly downregulated in both cancer types. This pattern suggests differential regulation of oxidative metabolism, with LSCC exhibiting stronger induction of oxidative phosphorylation genes relative to LUAD.

Genes involved in fatty acid metabolism demonstrated a mixed pattern. CPT1A was downregulated in LUAD but showed no significant change in LSCC, while FASN was reduced in both cancer types, contrasting with ACADVL, which was significantly upregulated in LUAD but unchanged in LSCC. The pentose phosphate pathway was consistently activated, with G6PD, TALDO1, and PGD all significantly overexpressed in LUAD and LSCC, reflecting enhanced NADPH production and nucleotide precursor synthesis.

One-carbon metabolism genes were also strongly induced, with MTHFD2 and MTHFD1 showing marked upregulation across both lung cancer subtypes, and SHMT1/2 demonstrating moderate increases. Similarly, nucleotide biosynthesis genes DHODH, RRM2, and TK1 were highly overexpressed in LUAD and LSCC, with RRM2 and TK1 showing some of the highest fold changes observed in this study.

Analysis of amino acid metabolism genes revealed significant upregulation of ASNS and MTHFD1 in both LUAD and LSCC, while PHGDH displayed a dual behavior, being slightly reduced in LUAD but strongly upregulated in LSCC. Genes regulating cellular redox balance also showed differential expression: NFE2L2 (NRF2) was downregulated in LUAD but upregulated in LSCC, GCLC was highly induced in both, while SOD2 and GPX4 exhibited variable patterns, with SOD2 reduced in both subtypes and GPX4 elevated in LUAD but slightly decreased in LSCC.

Autophagy- and ER stress–related genes were heterogeneously expressed. MAP1LC3B (LC3) and ATG7 were significantly downregulated in both LUAD and LSCC, indicating a suppression of basal autophagy, whereas DDIT3 (CHOP) and HSPA5 (BiP/GRP78) were significantly upregulated, consistent with activation of the ER stress response.

When extending the UALCAN-based analysis to BRCA, strong activation of anabolic pathways was observed, with FASN, PHGDH, and G6PD markedly upregulated, reflecting enhanced lipogenesis, serine biosynthesis, and pentose phosphate pathway activity. Nucleotide biosynthesis genes such as RRM2 and DHODH were also significantly elevated. In contrast, metastatic tumors in the MET500 dataset exhibited a broader induction of stress-related genes, including NFE2L2, SOD2, GPX4, DDIT3 (CHOP), HSPA5 (BiP), MAP1LC3B (LC3), and ATG7, consistent with enhanced redox balance, ER stress response, and autophagy activation.

**Fig 2:**
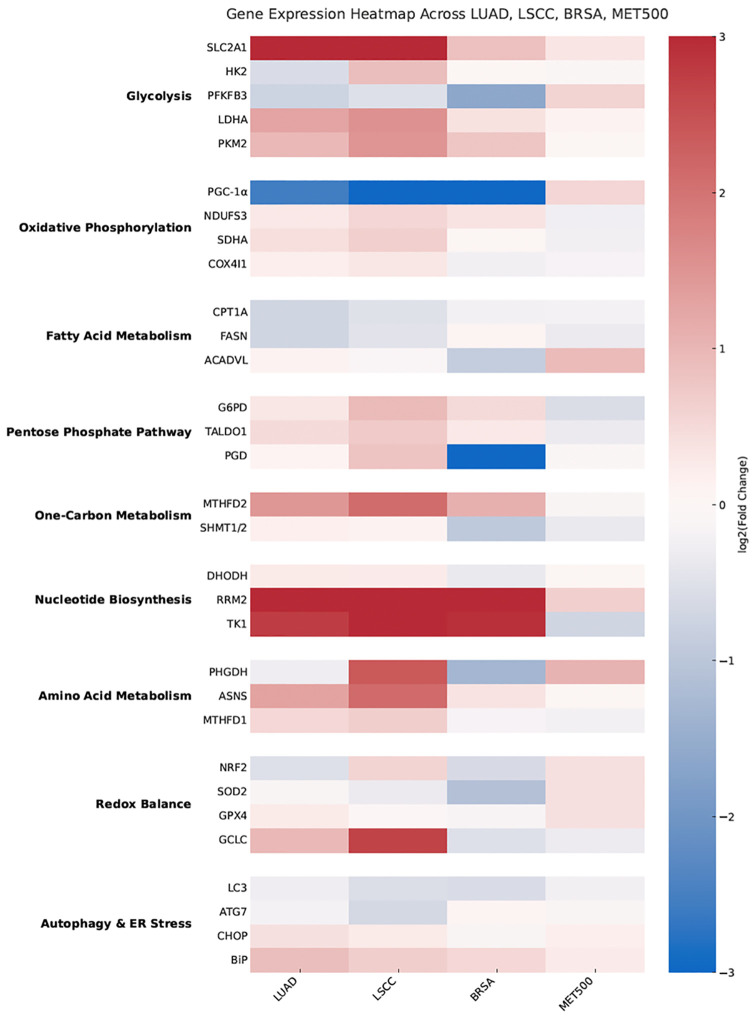
Differential expression of metabolic and stress-related genes across cancer types. Heat map representing log2 fold-change values of selected genes involved in glycolysis, oxidative phosphorylation, fatty acid metabolism, pentose phosphate pathway, one-carbon metabolism, nucleotide biosynthesis, amino acid metabolism, redox balance, and autophagy/ER stress in lung adenocarcinoma (LUAD), lung squamous cell carcinoma (LSCC), breast adenocarcinoma (BRCA), and metastatic tumors (MET500), obtained from the UALCAN database.

A detailed summary of these results, including mean expression values, fold changes, p-values, regulation status, and sample numbers for each gene, is provided in S1 Table. To visualize global expression trends, a heat map was generated using log₂ fold-change values obtained from UALCAN, which highlights pathway-level differences across LUAD, LSCC, BRCA, and MET500 (Fig 2). Collectively, these results demonstrate that LUAD is predominantly characterized by glycolytic reprogramming, LSCC by stronger reliance on oxidative metabolism and nucleotide synthesis, BRCA by anabolic and lipogenic activation, and MET500 by stress-adaptive metabolic remodeling.

### Clinically actionable drug–gene interactions

**Fig 3:**
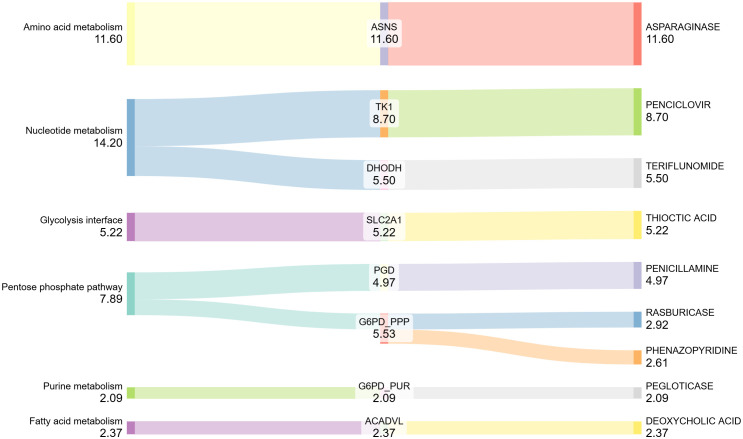
Sankey diagram showing FDA-approved drug–gene interactions (score ≥2.0) mapped to cancer-related metabolic pathways. Flow widths represent interaction scores retrieved from DGIdb.

After applying the inclusion criteria (FDA-approved drugs with interaction scores ≥ 2.0), nine high-confidence drug–gene interactions linked to cancer-associated metabolic pathways were identified from the initial list of 289 genes (S2 Table). A Sankey diagram was generated to visualize the flow from metabolic pathways to genes and their associated FDA-approved drugs (Fig 3). A detailed summary of these interactions is presented in Table 1.

**Table 1:** High-confidence FDA-approved drug–gene interactions (interaction score ≥2.0) identified via DGIdb.

Gene	Pathway	Drug	Regulatory approval	Interaction score
ASNS	Amino acid metabolism	ASPARAGINASE	Approved	11.60
TK1	Nucleotide metabolism	PENCICLOVIR	Approved	8.70
DHODH	Nucleotide metabolism	TERIFLUNOMIDE	Approved	5.50
SLC2A1	Glycolysis interface	THIOCTIC ACID	Approved	5.22
PGD	Pentose phosphate pathway	PENICILLAMINE	Approved	4.97
G6PD	Pentose phosphate pathway	RASBURICASE	Approved	2.92
G6PD	Pentose phosphate pathway	PHENAZOPYRIDINE	Approved	2.61
ACADVL	Fatty acid metabolism	DEOXYCHOLIC ACID	Approved	2.37
G6PD	Purine metabolism	PEGLOTICASE	Approved	2.09

In the amino acid metabolism pathway, ASNS showed the strongest interaction, with an interaction score of 11.60 linked to Asparaginase. In the nucleotide metabolism, TK1 and DHODH were connected to Penciclovir and Teriflunomide, with scores of 8.70 and 5.50, respectively. For central carbon metabolism, SLC2A1, positioned at the glycolysis interface, was found to interact with Thioctic acid (5.22).

In the pentose phosphate pathway, PGD was associated with Penicillamine (interaction score: 4.97), while G6PD showed interactions with Rasburicase (2.92) and Phenazopyridine (2.61). In the fatty acid metabolism pathway, ACADVL was linked to Deoxycholic acid (2.37). Lastly, in purine metabolism, G6PD demonstrated an additional interaction with Pegloticase (2.09).

### Survival analysis using UALCAN

Survival analysis of the selected genes (ASNS, TK1, PGD, G6PD, SLC2A1, ACADVL, and DHODH) using UALCAN revealed cancer type-specific associations with patient outcomes. In LUAD, three genes displayed significant prognostic relevance. SLC2A1 showed the strongest association, with high expression correlating with poorer overall survival (p = 0.0001). TK1 was also significantly associated with reduced survival (p = 0.011), while PGD exhibited a modest but significant association (p = 0.031). The remaining genes ASNS, DHODH, G6PD, and ACADVL did not demonstrate significant effects on LUAD survival (p > 0.05). In LSCC, none of the evaluated genes showed a statistically significant impact on survival, indicating limited prognostic utility within this subtype. In BRCA, only G6PD exhibited a significant association with poorer survival (p = 0.032), whereas TK1 showed a borderline effect (p = 0.053), and all other genes displayed non-significant associations (p > 0.1). No survival analysis could be performed for MET500 due to the absence of patient survival metadata.

### Identification of clinical trial evidence for DGIdb-identified drugs

No registered interventional clinical trials were identified for any of the DGIdb-identified candidate drugs across LUAD, LSCC, and BRCA. In contrast, within the MET500 context, teriflunomide and deoxycholic acid were each associated with registered studies in metastatic disease (NCT03709446, status: Active, not recruiting; and NCT06842472, status: Not yet recruiting, respectively) [20,21]. In addition, an asparaginase-based formulation (eryaspase) has been evaluated in metastatic or locally recurrent triple-negative breast cancer (NCT03674242, Phase 2/3, status: Terminated due to sponsor decision) [22].

### Literature-based experimental validation of bioinformatics findings

The literature-based experimental validation revealed variable levels of evidence supporting the anticancer relevance of DGIdb-identified drugs across LUAD, LSCC, BRCA, and MET500 models. For asparaginase, multiple in vitro studies in LUAD consistently demonstrated strong anticancer activity, including cell-cycle disruption, replication stress, potent cytotoxicity in A549 cells, and enhanced efficacy when delivered via nanoparticle-based approaches [23–25] Teriflunomide showed supportive evidence at both computational and experimental levels, being prioritized as a repurposed therapeutic candidate for LUAD through CRISPR co-essentiality network analysis, and demonstrating enhanced cytotoxicity, apoptosis induction, and improved pharmacokinetic profiles in TNBC models when formulated in nanoparticle systems [26,27].

Thioctic acid was supported by extensive experimental evidence in LUAD and BRCA, with studies reporting inhibition of tumor growth, induction of apoptosis, suppression of migration and invasion, increased oxidative stress, reduced anoikis resistance, and enhanced sensitivity to chemotherapeutic agents in vitro and in vivo [28–32]. Penicillamine also demonstrated anticancer activity across lung and breast cancer models, including selective cancer cell killing through oxidative stress mechanisms, inhibition of tumor growth and metastasis in vivo, reversal of hypoxia-induced epithelial–mesenchymal transition, and modulation of metabolic and immune-related pathways [33–35].

For deoxycholic acid, mixed effects were observed depending on cancer context. While deoxycholic acid–chalcone conjugates exhibited strong anticancer activity in LUAD cell lines, deoxycholic acid promoted proliferation in HER2-positive breast cancer cells, suggesting context-dependent and potentially tumor-promoting effects [36,37]. In contrast, no experimental evidence was identified for penciclovir, rasburicase, phenazopyridine, or pegloticase in the evaluated cancer types, indicating a lack of current experimental validation for these candidates within the selected contexts.

### Gene–disease associations across cancer types

Based on prior drug–gene interaction analysis, seven genes (ASNS, TK1, DHODH, SLC2A1, PGD, G6PD, and ACADVL) were shortlisted for further evaluation using the Open Targets Platform to assess their relevance across four cancer types: LUAD, LSCC, BRCA, and MET500. For each gene–disease pair, two metrics were collected: the Association Score, a normalized value (0–1) reflecting the overall strength of evidence from various sources, and the Europe PMC score, which specifically indicates literature-derived support based on text mining of peer-reviewed publications.

**Fig 4:**
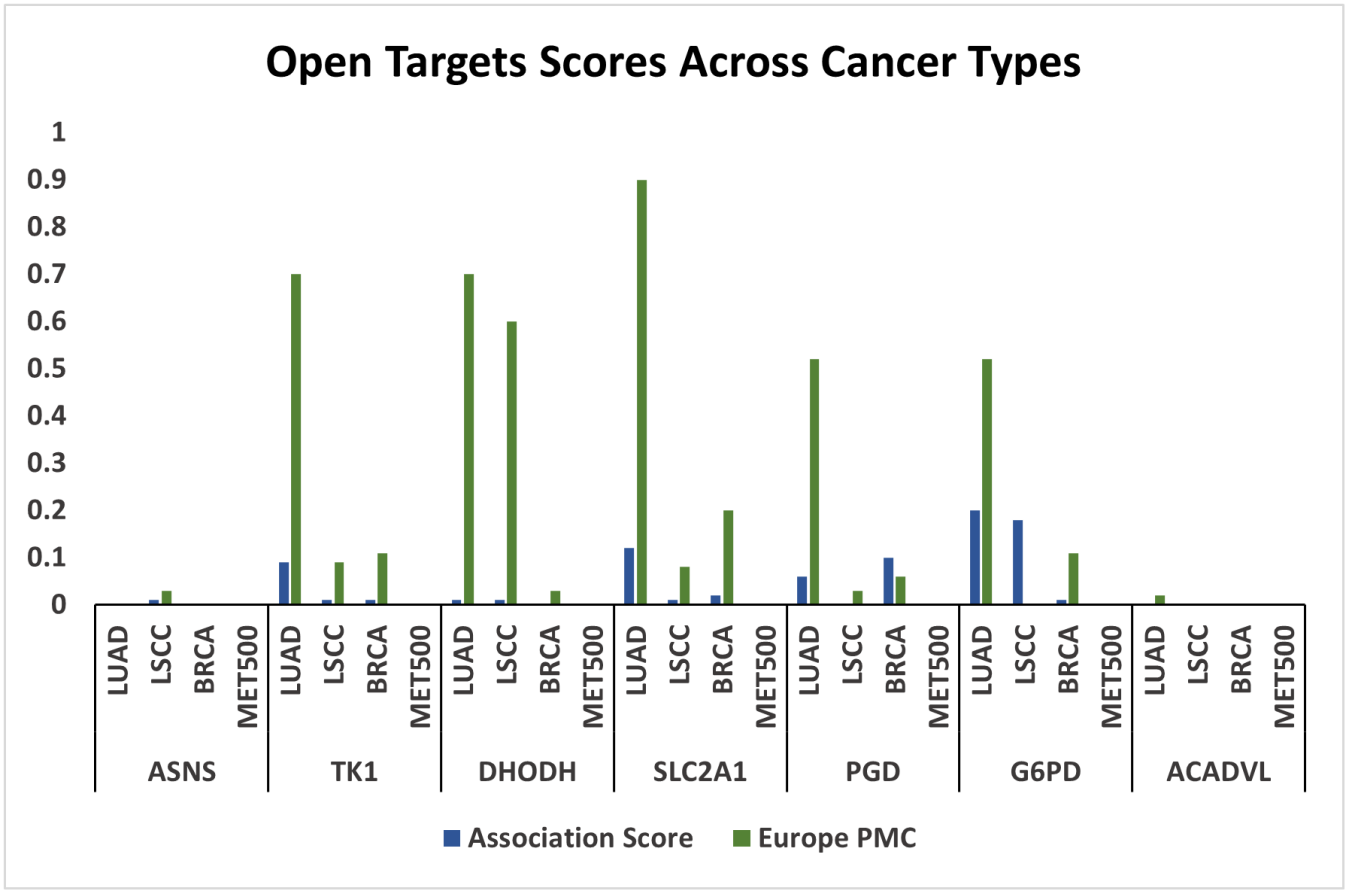
Association scores and literature-based evidence for selected genes across four cancer types using the Open Targets Platform. Association scores indicate the overall strength of evidence linking each gene to the respective cancer type (range: 0–1). Europe PMC scores reflect literature-derived support based on text mining of peer-reviewed publications.

As shown in Fig 4, G6PD exhibited the highest association scores with lung adenocarcinoma (0.20) and lung squamous cell carcinoma (0.18), followed by SLC2A1 with a notable association in LUAD (0.12) and strong literature support (0.9). TK1 also showed high Europe PMC evidence in LUAD (0.7). Conversely, ASNS and ACADVL lacked sufficient data across most cancer types. Notably, no association data were available for any of the selected genes in metastatic breast cancer (MET500). Overall, the Consistency between association scores and literature evidence supports the potential biological significance of these genes in the cancers investigated.

### Enriched KEGG pathways involving prioritized genes

KEGG pathway enrichment analysis was employed to identify biologically meaningful pathways enriched among the selected genes, providing insights into their potential functional roles in cancer metabolism [38]. Pathway enrichment analysis was performed using the KEGG 2021 Human database, resulting in a full list of enriched pathways (S3 Table). From this list, seven pathways were retained based on adjusted p-values < 0.05 and high combined scores. Selection criteria prioritized both statistical significance and biological relevance to cancer metabolism (Table 2).The pentose phosphate pathway (G6PD, PGD) showed the strongest enrichment (adjusted p = 8.18 × 10 ⁻ ⁴; combined score = 2851.75), highlighting its essential role in maintaining redox balance and supporting anabolic metabolism in cancer cells [39] Other highly enriched pathways included pyrimidine metabolism (TK1, DHODH) and glutathione metabolism (G6PD, PGD), both of which are involved in nucleotide synthesis and oxidative stress response. The pathway central carbon metabolism in cancer (G6PD, SLC2A1) further emphasized the metabolic reprogramming characteristic of tumor progression [40]. In addition, diabetic cardiomyopathy (G6PD, SLC2A1) and alanine, aspartate, and glutamate metabolism (ASNS) were significantly enriched, reflecting altered amino acid and glucose handling in cancer [41]. Lastly, fatty acid degradation (ACADVL) was also highlighted, consistent with the energy demands of rapidly proliferating tumor cells [42]. Collectively, these findings highlight the involvement of the selected genes in key metabolic pathways associated with cancer biology.

**Table 2:** The top enriched KEGG pathways identified from the selected gene set using Enrichr (full list available in S3 Table).

Pathway	Adjusted P-value	Odds Ratio	Combined Score	Genes
**Pentose phosphate pathway**	0.00082	285.21	2851.75	G6PD;PGD
**Pyrimidine metabolism**	0.00100	147.70	1290.68	TK1;DHODH
**Glutathione metabolism**	0.00100	145.00	1262.00	G6PD;PGD
**Central carbon metabolism in cancer**	0.00113	117.21	971.77	G6PD;SLC2A1
**Diabetic cardiomyopathy**	0.00749	39.39	243.20	G6PD;SLC2A1
**Alanine, aspartate, and glutamate metabolism**	0.03846	92.39	402.10	ASNS
**Fatty acid degradation**	0.03846	79.17	332.73	ACADVL

## Functional insights from GO biological process enrichment

This analysis aimed to gain functional insights into the biological roles of the selected genes, particularly in relation to cancer metabolism and cellular regulation [17]. The analysis identified several significantly enriched biological processes associated with the input gene set (S4 Table). From the full list, biological processes with adjusted p-values < 0.05 and high combined scores were retained based on their statistical significance and relevance to cancer-related metabolic reprogramming (Table 3).

**Table 3:** Top Enriched GO Biological Processes Identified by Enrichr Based on Cancer-Associated Gene Set (full list available in S4 Table).

Biological Process	Overlap	Adjusted P-value	Odds Ratio	Combined Score	Genes
L-ascorbic Acid Metabolic Process (GO:0019852)	45778	0.0193	832.88	5287.72	SLC2A1
Pyrimidine Ribonucleotide Metabolic Process (GO:0009218)	45778	0.0193	832.88	5287.72	DHODH
Monosaccharide Metabolic Process (GO:0005996)	45839	0.0193	555.19	3338.15	SLC2A1
Pyrimidine Ribonucleotide Biosynthetic Process (GO:0009220)	45839	0.0193	555.19	3338.15	DHODH
Negative Regulation of Fatty Acid Oxidation (GO:0046322)	45839	0.0193	555.19	3338.15	ACADVL
Mitotic DNA Replication (GO:1902969)	45931	0.0193	370.07	2093.27	TK1
Fatty Acid Beta-Oxidation Using acyl-CoA Dehydrogenase (GO:0033539)	45931	0.0193	370.07	2093.27	ACADVL
D-glucose Import (GO:0046323)	42370	0.0198	221.98	1151.45	SLC2A1
Positive Regulation of Cell Cycle (GO:0045787)	21916	0.0319	56.31	218.04	ASNS

The most enriched terms included L-ascorbic acid metabolic process and pyrimidine ribonucleotide metabolic process (adjusted p-value = 0.0193; odds ratio = 832.88), involving SLC2A1 and DHODH, respectively. Other enriched pathways included monosaccharide metabolic process and negative regulation of fatty acid oxidation, both with high combined scores (>3300), indicating strong enrichment. Notably, genes such as ACADVL, TK1, and ASNS were involved in processes related to fatty acid metabolism and cell cycle regulation, further suggesting functional relevance to cancer-related metabolic remodeling.

## Protein–protein interaction network reveals functional connectivity

To explore potential functional associations among the shortlisted metabolic genes (ASNS, TK1, DHODH, SLC2A1, PGD, G6PD, and ACADVL), protein–protein interaction (PPI) data were retrieved from the STRING database and visualized using Cytoscape. Interactions were based on the combined STRING confidence score, integrating evidence from experimental data, curated databases, and text mining. The full STRING interaction data, including evidence types and combined confidence scores, is provided in S5 Table.

**Fig 5:**
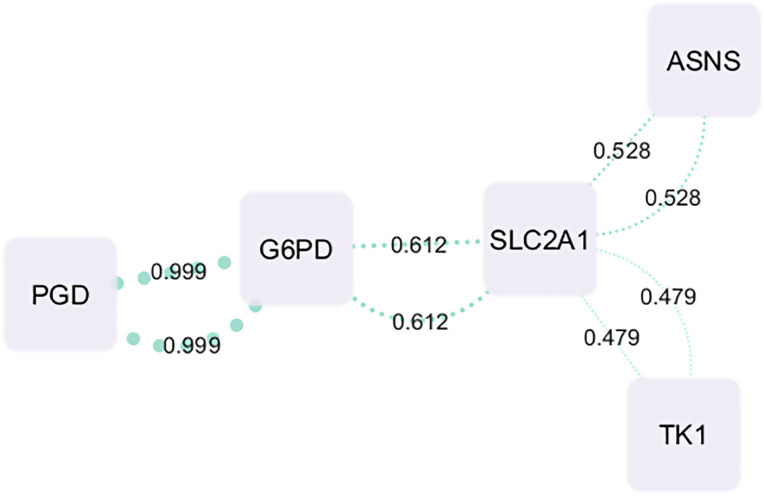
Protein–Protein Interaction Network of Selected Metabolic Genes. The interaction network of selected genes (G6PD, PGD, SLC2A1, ASNS, and TK1) was constructed using STRING database scores and visualized in Cytoscape. Edge thickness corresponds to the combined interaction score, reflecting confidence based on experimental evidence, databases, and text mining. G6PD shows strong interaction with PGD (score = 0.999), and SLC2A1 connects moderately with G6PD, ASNS, and TK1.

According to Fig 5, the resulting PPI network included five genes with reported interactions: G6PD, PGD, SLC2A1, ASNS, and TK1. G6PD appeared as a central node, showing a strong interaction with PGD (combined score = 0.999). It also exhibited a moderate association with SLC2A1 (0.612). SLC2A1 was further linked to ASNS and TK1 with scores of 0.528 and 0.479, respectively. No interaction data were found for DHODH and ACADVL under the applied confidence threshold. The network highlights a potential metabolic cluster centered on G6PD and PGD, suggesting co-regulation or functional interplay in cancer-related metabolic pathways.

## Discussion

Our integrative analysis revealed that cancer metabolism is not a uniform process but rather exhibits striking cancer type–specific rewiring, with LUAD, LSCC, BRCA, and MET500 each adopting distinct metabolic programs that shape their progression and therapeutic vulnerabilities.

In this study, we systematically profiled metabolic gene expression across LUAD, LSCC, BRCA, and metastatic tumors using UALCAN-based transcriptomic analysis, revealing both conserved and context-specific signatures. Glycolysis emerged as a dominant feature of LUAD, reflected in the robust upregulation of SLC2A1, LDHA, and PKM2. This is consistent with the classical Warburg effect, where tumors shift toward aerobic glycolysis even under oxygen-replete conditions to support anabolic demands [43,44]. Upregulation of SLC2A1 has been associated with poor prognosis in non-small cell lung cancer and linked to resistance to targeted therapies, and correlated with a poorer prognosis [45]. Interestingly, while glycolysis was strongly induced in LUAD, LSCC showed a more balanced metabolic program with simultaneous activation of oxidative phosphorylation genes, including NDUFS3, SDHA, and COX4I1. This dual reliance on glycolysis and mitochondrial respiration suggests metabolic flexibility, which has been previously described as a survival advantage for squamous tumors [46]

Fatty acid metabolism showed a less uniform pattern. The downregulation of CPT1A in LUAD may indicate reduced fatty acid oxidation, a shift that favors glycolysis and lipogenesis for biomass production. On the other hand, ACADVL was elevated, suggesting heterogeneity in lipid utilization within tumor subsets. Previous studies have linked CPT1A inhibition to impaired tumor growth and metastasis [46], while ACADVL expression has been implicated in sustaining mitochondrial metabolism under nutrient stress [47]

The pentose phosphate pathway (PPP) was consistently activated in LUAD and LSCC, as demonstrated by elevated G6PD, TALDO1, and PGD. This pathway generates NADPH to maintain redox balance and supplies ribose-5-phosphate for nucleotide biosynthesis. Hyperactivation of the PPP has been recognized as a hallmark of chemoresistance and tumor survival under oxidative stress [47,48]. Notably, PPP upregulation converges with our enrichment results, underscoring its importance as a metabolic hub in these tumors.

One-carbon metabolism genes, particularly MTHFD2 and MTHFD1, were strongly induced, aligning with prior reports that link these enzymes to increased nucleotide synthesis and aggressive tumor phenotypes [49]. Similarly, the dramatic upregulation of nucleotide biosynthesis genes such as RRM2 and TK1 is in agreement with the high proliferative drive of cancer cells [50]. Elevated RRM2 has been associated with poor survival in lung and breast cancers [51,52], while TK1 has long been used as a serum biomarker of tumor burden and therapeutic response [53].

Amino acid metabolism revealed additional cancer-specific adaptations. ASNS was significantly induced, consistent with its role in supporting metabolic adaptation under nutrient limitation and with clinical relevance in acute lymphoblastic leukemia treated with asparaginase [54]. The divergent regulation of PHGDH between LUAD (downregulated) and LSCC (upregulated) is particularly interesting, as PHGDH is a rate-limiting enzyme in serine biosynthesis. Its overexpression has been linked to enhanced nucleotide synthesis and poor prognosis in triple-negative breast cancer [55], suggesting that LSCC may rely more heavily on serine metabolism than LUAD.

Redox and stress pathways further distinguished cancer types. NFE2L2 (NRF2) was suppressed in LUAD but induced in LSCC, reflecting different oxidative stress management strategies. NRF2 activation is well established as a driver of chemoresistance and metabolic adaptation [56]. Consistent with this, GCLC, a key enzyme in glutathione biosynthesis, was highly induced, while SOD2 was reduced, suggesting an imbalance in antioxidant defenses. Autophagy- and ER stress–related genes also revealed adaptive programs: HSPA5 (BiP) and DDIT3 (CHOP) were upregulated, highlighting reliance on unfolded protein response pathways that protect tumor cells under proteotoxic stress [57–59].

When comparing primary BRCA to metastatic MET500 tumors, distinct metabolic rewiring was evident. BRCA was characterized by lipogenic and anabolic activation, exemplified by increased FASN, PHGDH, and G6PD. By contrast, metastatic tumors displayed a broader stress-adaptive program, with coordinated upregulation of antioxidant and autophagy-related genes (NFE2L2, GPX4, SOD2, MAP1LC3B, ATG7). This shift toward stress management mirrors observations in metastatic disease, where adaptation to oxidative stress and immune surveillance is critical for colonization and survival in distant tissues [60].

Beyond expression, drug–gene interaction analysis identified multiple metabolic enzymes with connections to FDA-approved drugs. For example, ASNS was strongly linked to asparaginase, a drug long used in acute lymphoblastic leukemia, raising the possibility that asparagine dependency could extend to subsets of solid tumors [61]. DHODH, upregulated in both LUAD and LSCC, was linked to teriflunomide, a pyrimidine synthesis inhibitor currently under investigation for anticancer activity [62]. Similarly, TK1 was connected to penciclovir, a nucleoside analog with potential anti-proliferative applications [63]. SLC2A1, the principal glucose transporter, was associated with thioctic acid (α-lipoic acid), which has antioxidant and metabolic modulatory properties [64]. These findings emphasize that known drugs, including that outside traditional oncology, may be repurposed to target cancer metabolic vulnerabilities.

Gene–disease association profiling using Open Targets further contextualized these genes. G6PD and SLC2A1 emerged with the strongest evidence for association with LUAD and LSCC, reflecting their well-established roles in redox regulation and glucose uptake in lung tumors [65,66]. Interestingly, ASNS and ACADVL displayed minimal evidence in cancer association databases, suggesting that while they are functionally altered, they remain underexplored in the clinical literature.

At the systems level, KEGG pathway enrichment revealed a consistent prioritization of the pentose phosphate pathway, pyrimidine metabolism, and glutathione metabolism, all of which are central to supporting redox balance and nucleotide biosynthesis in proliferating cells [67–69]. The strong enrichment of the PPP, driven by G6PD and PGD, aligns with prior work linking PPP activation to chemoresistance and metastatic progression [70]. Pyrimidine metabolism, represented by DHODH and TK1, shows the nucleotide dependency of rapidly dividing tumor cells, while glutathione metabolism links metabolic reprogramming to oxidative stress defense and ferroptosis evasion [71]. GO enrichment analysis further reinforced these themes, highlighting terms such as pyrimidine ribonucleotide biosynthesis, D-glucose import, and mitotic DNA replication, integrating metabolic remodeling directly with proliferative and stress-regulatory functions [72].

Finally, PPI network analysis revealed a central metabolic hub centered on G6PD and PGD, with strong interactions suggesting co-regulation of the PPP as a dominant cancer node. SLC2A1 linked this PPP module with amino acid metabolism (ASNS) and nucleotide metabolism (TK1), creating a cross-pathway axis of glucose uptake, redox balance, and nucleotide biosynthesis. Such integrative network findings highlight how metabolic reprogramming is not confined to isolated pathways but instead forms interconnected modules that collectively sustain tumor growth and survival.

To validate the bioinformatics-derived findings, a layered validation strategy integrating patient outcomes, clinical trial activity, and literature evidence was applied. Survival analysis showed that several prioritized metabolic genes were prognostically relevant, supporting the clinical significance of the identified metabolic alterations. This convergence between molecular dysregulation and survival outcomes supports the biological relevance of the bioinformatics-based prioritization.

Clinical trial screening was used to assess the translational progression of the computationally identified drug–gene interactions. The limited number of completed or advanced-phase trials reveals a substantial gap between in silico target nomination and clinical implementation. Consequently, many prioritized candidates have not yet been systematically evaluated in clinical settings.

Finally, to assess the extent of the gap between bioinformatic predictions and experimental validation, a targeted literature assessment was conducted. This analysis revealed that several metabolism-targeting agents have already been examined in preclinical cancer models, whereas others remain largely unexplored in relevant oncological contexts. The identification of both previously investigated and untested candidates demonstrates that the bioinformatics framework can recover biologically established targets while simultaneously nominating novel therapeutic opportunities. Moreover, the presence of ongoing experimental and translational research for a subset of the prioritized drug candidates suggests that this bioinformatics-driven approach can be effectively applied before experimental or clinical investigation to guide hypothesis generation, refine target selection, and inform study design in the field of cancer metabolism.

## Conclusions

This multi-layered analysis provides a comprehensive view of metabolic remodeling across lung adenocarcinoma, lung squamous cell carcinoma, breast cancer, and metastatic breast tumors, highlighting clear cancer type–specific metabolic programs. Lung adenocarcinoma was primarily characterized by enhanced glycolysis and pentose phosphate pathway activity, lung squamous cell carcinoma showed a mixed glycolytic–oxidative phenotype, breast cancer exhibited increased anabolic and lipogenic metabolism, and metastatic breast tumors displayed prominent stress-response and autophagy-related features.

By integrating transcriptomic profiling with drug–gene interaction mapping, pathway enrichment, survival analysis, clinical trial screening, and literature-based evaluation, we placed these metabolic alterations within a functional and clinical context. This approach reinforced known metabolic hallmarks, such as glycolytic reprogramming in LUAD and anabolic activation in BRCA, while also identifying underexplored vulnerabilities, including ASNS- and ACADVL-associated pathways. Several prioritized metabolic enzymes, including ASNS, DHODH, TK1, and G6PD, were linked to FDA-approved drugs, indicating potential opportunities for therapeutic repurposing. Moreover, survival analyses demonstrated that altered expression of selected metabolic genes, such as SLC2A1, TK1, and G6PD, was associated with patient outcomes in specific cancer types, supporting their clinical relevance.

Although direct functional experiments were not performed, the layered validation strategy applied here demonstrates that integrating survival outcomes with clinical and literature-based evidence can corroborate bioinformatics-driven predictions. Overall, this study provides a structured framework for prioritizing metabolically relevant targets and pathways, and highlights the need for future experimental validation and early-phase clinical studies to translate computational discoveries into actionable therapeutic strategies in cancer metabolism.

## Supporting information

S1 Table Gene expression full. (DOCX)

S2 Table Full list of 289 analyzed genes with corresponding drug gene interaction scores from DGIdb.docx. (DOCX)

S3 Table Full List of KEGG Pathways Enriched from the Selected Gene Set Using Enrichr. (DOCX)

S4 Table Full List of GO Biological Processes Enriched from the Selected Gene Set Using Enrichr. (DOCX)

S5 Table Full STRING Protein–Protein Interaction Data for the Selected Gene Set. (DOCX)

## Abbreviations

ACADVL: Acyl-CoA dehydrogenase, very long chain
ASNS: Asparagine synthetase
ATP: Adenosine triphosphate
BRCA: Breast cancer
COX4I1: Cytochrome c oxidase subunit 4 isoform 1
DGIdb: Drug–Gene Interaction Database
DHODH: Dihydroorotate dehydrogenase
DNA: Deoxyribonucleic acid
EMT: Epithelial–mesenchymal transition
ER: Endoplasmic reticulum
FASN: Fatty acid synthase
FDA: U.S. Food and Drug Administration
G6PD: Glucose-6-phosphate dehydrogenase
GO: Gene Ontology
GPX4: Glutathione peroxidase 4
HSPA5 (BiP/GRP78): Heat shock protein family A member 5
KEGG: Kyoto Encyclopedia of Genes and Genomes
LDHA: Lactate dehydrogenase A
LUAD: Lung adenocarcinoma
LSCC: Lung squamous cell carcinoma
MAP1LC3B (LC3): Microtubule-associated protein 1 light chain 3 beta
MET500: Metastatic breast tumors dataset
MTHFD1/2: Methylenetetrahydrofolate dehydrogenase 1/2
NADPH: Nicotinamide adenine dinucleotide phosphate (reduced)
NFE2L2 (NRF2): Nuclear factor erythroid 2–related factor 2
NSCLC: Non-small cell lung cancer
Open Targets: Open Targets Platform
PFKFB3: 6-Phosphofructo-2-kinase/fructose-2,6-bisphosphatase 3
PGC-1α (PPARGC1A): Peroxisome proliferator-activated receptor gamma coactivator 1-alpha
PGD: Phosphogluconate dehydrogenase
PHGDH: Phosphoglycerate dehydrogenase
PKM2: Pyruvate kinase isoform M2
PPI: Protein–protein interaction
PPP: Pentose phosphate pathway
RRM2: Ribonucleotide reductase regulatory subunit M2
SCD1: Stearoyl-CoA desaturase 1
SDHA: Succinate dehydrogenase complex flavoprotein subunit A
SLC2A1 (GLUT1): Solute carrier family 2, member 1 (glucose transporter 1)
SOD2: Superoxide dismutase 2
STRING: Search Tool for the Retrieval of Interacting Genes/Proteins
STAD: Stomach adenocarcinoma
TCGA: The Cancer Genome Atlas
TALDO1: Transaldolase 1
TK1: Thymidine kinase 1
UALCAN: University of Alabama at Birmingham Cancer Data Analysis Portal
WHO: World Health Organization
