# Supplementary material for: Mapping metabolic reprogramming in lung and breast cancer through integrative bioinformatics
Source: PLoS One. 2026 Jun 4;21(6):e0350628. doi: 10.1371/journal.pone.0350628 (PMC13235884; doi:10.1371/journal.pone.0350628)
Supplement: S1 Table — (DOCX) [file pone.0350628.s001.docx]

| Pathway | Gene | Function | Relevance in cancer | LC (**LUAD**) expression (mean, tumor vs. normal) | Fold change | Statistical significance (p-value) | Up or down regulated | Number of samples (tumor vs. normal) | LC (**LSCC**) expression (tumor vs. normal) | Fold change | Statistical significance (p-value) | Up or down regulated | Number of samples (tumor vs. normal) |  |
| --- | --- | --- | --- | --- | --- | --- | --- | --- | --- | --- | --- | --- | --- | --- |
| **Glycolysis** | **SLC2A1** | Glucose transporter | Upregulated in highly glycolytic tumors (Warburg effect) | 44.109 vs. 5.269 | 8.37 folds | **1.62447832963153E-12** | Upregulated | 515 vs. 59 | 356.497 vs. 6.731 | 52.96 folds | **1.62447832963153E-12** | Upregulated | 503 vs. 52 |  |
|  | **HK2** | Hexokinase 2 | Catalyses first step of glycolysis; linked to chemoresistance | 14.449 vs. 21.696 | 0.665 folds | 7.299900E-02 | Insignificant change |  | 36.473 vs.19.37 | 1.88 | **1.62447832963153E-12** | Upregulated |  |  |
|  | **PFKFB3** | Phosphofructokinase-2 | Regulates glycolytic flux; high in aggressive tumors | 49.187 vs.83.562 | 0.588 folds | **1.361330E-03** | Downregulated |  | 46.912 vs. 68.211 | 0.69 | **5.60949999999227E-05** | Downregulated |  |  |
|  | **LDHA** | Lactate dehydrogenase | Converts pyruvate to lactate; linked to metastasis | 592.976 vs. 242.99 | 2.44 folds | **1.62447832963153E-12** | Upregulated |  | 720.518 vs. 247.737 | 2.91 | **<1E-12** | Upregulated |  |  |
|  | **PKM2** | Pyruvate kinase M2 | Tumor-specific isoform that promotes aerobic glycolysis | 971.349 vs. 497.927 | 1.950 | **1.62447832963153E-12** | Upregulated |  | 1451.628 vs. 514.862 | 2.82 | **1.62436730732907E-12** | Upregulated |  |  |
| **Oxidative Phosphorylation (OXPHOS)** | **PGC-1α (PPARGC1A)** | Mitochondrial biogenesis regulator | mitochondrial ATP production, supporting metastasis | 0.562 vs. 3.389 | 0.17 | **7.592400E-04** | Downregulated |  | 0.368 vs. 3.101 | 0.12 | **3.41615624677161E-13** | Downregulated |  |  |
|  | **NDUFS3** | Mitochondrial complex I subunit | Regulates oxidative phosphorylation | 109.385 vs. 88.704 | 1.23 | **<1E-12** | Upregulated |  | 123.884 vs. 84.499 | 1.47 | **<1E-12** | Upregulated |  |  |
|  | **SDHA** | Succinate dehydrogenase | Involved in the TCA cycle and electron transport chain | 81.284 vs. 59.714 | 1.36 | **1.62458935193399E-12** | Upregulated |  | 98.913 vs. 63.517 | 1.56 | **<1E-12** | Upregulated |  |  |
|  | **COX4I1** | Cytochrome C oxidase subunit | Involved in mitochondrial respiration | 530.159 vs. 459.329 | 1.15 | **1.33110000000158E-06** | Upregulated |  | 573.713 vs. 462.922 | 1.24 | **1.62903024403249E-12** | Upregulated |  |  |
| **Fatty Acid Metabolism** | **CPT1A** | Carnitine palmitoyltransferase 1A | Controls fatty acid oxidation (FAO) | 24.169 vs. 39.817 | 0.61 | **6.25509999352403E-09** | Downregulated |  | 27.336 vs 38.782 | 0.70 | 2.280200E-01 | Insignificant change |  |  |
|  | **FASN** | Fatty acid synthase | Involved in de novo lipogenesis, essential for tumor growth | 66.983 vs. 109.544 | 0.61 | 1.611030E-01 | Insignificant change |  | 77.629 vs. 107.666 | 0.72 | **8.101100E-03** | Downregulated |  |  |
|  | **SCD1** | No data | | | | |  |  |  |  |  |  |  | |
|  | **ACADVL** | Very long-chain acyl-CoA dehydrogenase | Controls fatty acid breakdown | 154.488 vs. 141.615 | 1.09 | **3.8681999825485E-09** | Upregulated |  | 133.808 vs. 144.014 | 0.93 | 7.067400E-01 | Insignificant change |  |  |
| **Pentose Phosphate Pathway (PPP)** | **G6PD** | Glucose-6-phosphate dehydrogenase | Produces NADPH, protecting against oxidative stress | 56.079 vs. 44.911 | 1.25 | **1.6242562850266E-12** | Upregulated |  | 96.048 vs. 49.494 | 1.94 | 1.62436730732907E-12 | Upregulated |  |  |
|  | **TALDO1** | Transaldolase | Regulates ribose-5-phosphate production for nucleotide synthesis | 263.278 vs. 187.724 | 1.40 | **1.62447832963153E-12** | Upregulated |  | 317.31 vs. 193.942 | 1.64 | **1.62447832963153E-12** | Upregulated |  |  |
|  | **PGD** | 6-Phosphogluconate dehydrogenase | Involved in ribulose-5-phosphate generation | 137.707 vs. 128.293 | 1.07 | **1.94733118519252E-12** | Upregulated |  | 241.137 vs. 138.006 | 1.75 | **<1E-12** | Upregulated |  |  |
| **One-Carbon Metabolism** | **MTHFD2** | Methylenetetrahydrofolate dehydrogenase | Supports nucleotide synthesis, highly expressed in tumors | 49.325 vs. 17.865 | 2.76 | **<1E-12** | Upregulated |  | 89.634 vs. 20.859 | 4.30 | **<1E-12** | Upregulated |  |  |
|  | **SHMT1/2** | Serine hydroxymethyltransferase | Key for folate metabolism and purine synthesis | 22.252 vs. 19.699 | 1.13 | **1.62458935193399E-12** | Upregulated |  | 19.884 vs. 18.112 | 1.10 | **1.18679954752565E-10** | Upregulated |  |  |
| **Nucleotide Biosynthesis** | **DHODH** | Dihydroorotate dehydrogenase | Required for pyrimidine biosynthesis | 6.712 vs. 5.531 | 1.21 | **1.62447832963153E-12** | Upregulated |  | 6.983 vs. 5.851 | 1.19 | **2.59420263049037E-11** | Upregulated |  |  |
|  | **RRM2** | Ribonucleotide reductase subunit | Produces deoxyribonucleotides for DNA synthesis | 18.004 vs. 1.958 | 9.18 | **1.62447832963153E-12** | Upregulated |  | 42.405 vs. 1.942 | 21.84 | **<1E-12** | Upregulated |  |  |
|  | **TK1** | Thymidine kinase | Important in DNA repair and replication | 52.236 vs. 7.684 | 6.79 | **1.62447832963153E-12** | Upregulated |  | 117.053 vs. 7.546 | 15.51 | **<1E-12** | Upregulated |  |  |
| **Amino Acid Metabolism** | **PHGDH** | Phosphoglycerate dehydrogenase | Controls serine biosynthesis, fueling nucleotide synthesis | 15.206 vs. 18.876 | 0.81 | **2.46169751250136E-11** | Downregulated |  | 87.396 vs. 16.826 | 5.19 | **1.11022302462516E-16** | Upregulated |  |  |
|  | **ASNS** | Asparagine synthetase | Critical for protein synthesis and metabolic adaptation | 25.578 vs. 10.402 | 2.46 | **<1E-12** | Upregulated |  | 46.621 vs. 10.6689 | 4.37 | **<1E-12** | Upregulated |  |  |
|  | **MTHFD1** | Methylenetetrahydrofolate dehydrogenase 1 | Important in amino acid and one-carbon metabolism | 29.796 vs. 20.479 | 1.45 | **1.62447832963153E-12** | Upregulated |  | 34.341 vs. 21.493 | 1.60 | **1.62447832963153E-12** | Upregulated |  |  |
| **Redox Balance & Detoxification** | **NRF2 (NFE2L2)** | Master regulator of oxidative stress response | Upregulated in chemo-resistant tumors | 74.561 vs. 107.887 | 0.69 | **1.83799997444112E-09** | Downregulated |  | 153.122 vs. 102.829 | 1.49 | **<1E-12** | Upregulated |  |  |
|  | **SOD2** | Superoxide dismutase | Neutralizes superoxide radicals | 363.757 vs. 396.494 | 0.92 | 1.482360E-01 | Downregulated |  | 423.706 vs. 538.191 | 0.79 | **7.918900E-03** | Downregulated |  |  |
|  | **GPX4** | Glutathione peroxidase | Regulates ferroptosis (iron-dependent cell death) | 366.185 vs. 301.633 | 1.21 | **2.22044604925031E-16** | Upregulated |  | 299.816 vs. 303.531 | 0.99 | **2.342800E-03** | Downregulated |  |  |
|  | **GCLC** | Glutamate-cysteine ligase | Rate-limiting enzyme in glutathione synthesis | 17.881 vs. 9.177 | 1.95 | **1.62436730732907E-12** | Upregulated |  | 58.339 vs. 9.103 | 6.41 | **1.62436730732907E-12** | Upregulated |  |  |
| **Autophagy & ER Stress** | **LC3 (MAP1LC3B)** | Autophagy marker | Key for autophagosome formation | 63.601 vs. 77.727 | 0.82 | **8.88980000723905E-11** | Downregulated |  | 54.726 vs. 80.087 | 0.68 | **1.62447832963153E-12** | Downregulated |  |  |
|  | **ATG7** | Autophagy-related protein 7 | Essential for autophagy initiation | 11.175 vs. 12.887 | 0.87 | **4.71650000000157E-06** | Downregulated |  | 8.761 vs. 13.89 | 0.63 | **1.62436730732907E-12** | Downregulated |  |  |
|  | **CHOP (DDIT3)** | ER stress-induced apoptosis marker | Upregulated during severe ER stress | 34.679 vs. 26.27 | 1.32 | **1.62481139653892E-12** | Upregulated |  | 50.951 vs. 42.244 | 1.21 | **<1E-12** | Upregulated |  |  |
|  | **BiP (GRP78, HSPA5)** | ER chaperone | Protects cancer cells from ER stress | 396.309 vs. 210.404 | 1.88 | **1.62458935193399E-12** | Upregulated |  | 346.884 vs. 217.038 | 1.60 | **1.62447832963153E-12** | Upregulated |  |  |

LUAD: Lung adenocarcinoma

LSCC: Lung squamous cell carcinoma

| Pathway | Gene | Function | Relevance in cancer | BC (**BRSA**) expression (mean, tumor vs. normal) | Fold change | Statistical significance (p-value) | Up or downregulated | Number of samples (tumor vs. normal) | BC (**MET500**) expression (without MYC amplification vs. with) | Fold change | Statistical significance (p-value) | Up or downregulated | Number of samples (without MYC amplification vs. with) |
| --- | --- | --- | --- | --- | --- | --- | --- | --- | --- | --- | --- | --- | --- |
| **Glycolysis** | **SLC2A1** | Glucose transporter | Upregulated in highly glycolytic tumors (Warburg effect) | 39.306 vs. 21.786 | 1.804 | **<1E-12** | Upregulated | 1097 vs. 114 | 28.879 vs. 22.906 | 1.261 | 4.980800E-01 | Insignificant change | 79 vs. 13 |
|  | **HK2** | Hexokinase 2 | Catalyses first step of glycolysis; linked to chemoresistance | 21.298 vs. 20.975 | 1.015 | 7.825200E-01 | Insignificant change |  | 11.465 vs. 12.223 | 0.938 | 9.839800E-01 | Insignificant change |  |
|  | **PFKFB3** | Phosphofructokinase-2 | Regulates glycolytic flux; high in aggressive tumors | 42.134 vs. 130.267 | 0.323 | **1.74427139398858E-12** | Downregulated |  | 14.497 vs. 9.785 | 1.482 | 2.230600E-01 | Insignificant change |  |
|  | **LDHA** | Lactate dehydrogenase | Converts pyruvate to lactate; linked to metastasis | 477.489 vs. 367.957 | 1.299 | **<1E-12** | Upregulated |  | 220.475 vs. 201.027 | 1.097 | 4.653200E-01 | Insignificant change |  |
|  | **PKM2** | Pyruvate kinase M2 | Tumor-specific isoform that promotes aerobic glycolysis | 840.019 vs. 483.192 | 1.737 | **<1E-12** | Upregulated |  | No data |  | **<1E-12** | Insignificant change |  |
| **Oxidative Phosphorylation (OXPHOS)** | **PGC-1α (PPARGC1A)** | Mitochondrial biogenesis regulator | mitochondrial ATP production, supporting metastasis | 0.209 vs. 1.667 | 0.125 | **1.1338000005523E-07** | Downregulated |  | 0.177 vs. 0.121 | 1.463 | 8.707800E-01 | Insignificant change |  |
|  | **NDUFS3** | Mitochondrial complex I subunit | Regulates oxidative phosphorylation | 125.52 vs. 97.054 | 1.292 | **1.62447832963153E-12** | Upregulated |  | 17.589 vs. 21.068 | 0.835 | 4.483600E-01 | Insignificant change |  |
|  | **SDHA** | Succinate dehydrogenase | Involved in the TCA cycle and electron transport chain | 81.203 vs. 80.419 | 1.010 | 6.701900E-02 | Insignificant change |  | 43.152 vs. 51.484 | 0.838 | 3.051000E-01 | Insignificant change |  |
|  | **COX4I1** | Cytochrome C oxidase subunit | Involved in mitochondrial respiration | 448.123 vs. 532.232 | 0.842 | **8.255400E-03** | Downregulated |  | 128.12 vs. 144.151 | 0.889 | 7.810000E-01 | Insignificant change |  |
| **Fatty Acid Metabolism** | **CPT1A** | Carnitine palmitoyltransferase 1A | Controls fatty acid oxidation (FAO) | 36.077 vs. 42.096 | 0.857 | **6.763300E-03** | Downregulated |  | 13.293 vs. 15.188 | 0.875 | 9.795000E-01 | Insignificant change |  |
|  | **FASN** | Fatty acid synthase | Involved in de novo lipogenesis, essential for tumor growth | 156.575 vs. 146.989 | 1.064 | 5.638600E-01 | Insignificant change |  | 94.691 vs. 119.424 | 0.793 | 6.386600E-01 | Insignificant change |  |
|  | **SCD1** | No data available | | | | |  |  |  |  |  |  |  |
|  | **ACADVL** | Very long-chain acyl-CoA dehydrogenase | Controls fatty acid breakdown | 134.325 vs. 248.029 | 0.541 | **<1E-12** | Downregulated |  | 161.778 vs. 85.215 | 1.898 | 3.306800E-01 | Insignificant change |  |
| **Pentose Phosphate Pathway (PPP)** | **G6PD** | Glucose-6-phosphate dehydrogenase | Produces NADPH, protecting against oxidative stress | 41.769 vs. 29.404 | 1.419 | **9.9724999999995E-05** | Upregulated |  | 19.724 vs. 29.303 | 0.673 | 8.011400E-01 | Insignificant change |  |
|  | **TALDO1** | Transaldolase | Regulates ribose-5-phosphate production for nucleotide synthesis | 231.299 vs. 188.995 | 1.223 | **2.22044604925031E-16** | Upregulated |  | 110.827 vs. 141.264 | 0.785 | 2.736600E-01 | Insignificant change |  |
|  | **PGD** | 6-Phosphogluconate dehydrogenase | Involved in ribulose-5-phosphate generation | 11.547 vs. 116.033 | 0.099 | 4.007000E-01 | Insignificant change |  | 62.307 vs. 65.495 | 0.951 | 6.986500E-02 | Insignificant change |  |
| **One-Carbon Metabolism** | **MTHFD2** | Methylenetetrahydrofolate dehydrogenase | Supports nucleotide synthesis, highly expressed in tumors | 68.18 vs. 31.899 | 2.137 | **<1E-12** | Upregulated |  | 22.839 vs. 25.06 | 0.911 | 9.919400E-01 | Insignificant change |  |
|  | **SHMT1/2** | Serine hydroxymethyltransferase | Key for folate metabolism and purine synthesis | 20.846 vs. 40.039 | 0.521 | **1.62436730732907E-12** | Downregulated |  | 9.392 vs. 12.094 | 0.777 | 8.143000E-01 | Insignificant change |  |
| **Nucleotide Biosynthesis** | **DHODH** | Dihydroorotate dehydrogenase | Required for pyrimidine biosynthesis | 5.071 vs. 6.563 | 0.773 | **9.71250000000312E-05** | Downregulated |  | 2.141 vs. 2.089 | 1.025 | 4.749200E-01 | Insignificant change |  |
|  | **RRM2** | Ribonucleotide reductase subunit | Produces deoxyribonucleotides for DNA synthesis | 20.368 vs. 1.697 | 12.0 | **<1E-12** | Upregulated |  | 20.655 vs. 13.247 | 1.559 | 2.495800E-01 | Insignificant change |  |
|  | **TK1** | Thymidine kinase | Important in DNA repair and replication | 54.109 vs. 7.185 | 7.54 | **1.62447832963153E-12** | Upregulated |  | 38.285 vs. 62.405 | 0.613 | 1.126200E-01 | Insignificant change |  |
| **Amino Acid Metabolism** | **PHGDH** | Phosphoglycerate dehydrogenase | Controls serine biosynthesis, fueling nucleotide synthesis | 37.511 vs. 93.854 | 0.400 | 8.878600E-01 | Insignificant change |  | 21.926 vs. 10.565 | 2.075 | 7.460000E-02 | Insignificant change |  |
|  | **ASNS** | Asparagine synthetase | Critical for protein synthesis and metabolic adaptation | 22.592 vs. 17.564 | 1.287 | **1.62447832963153E-12** | Upregulated |  | 12.526 vs. 12.326 | 1.016 | 3.915600E-01 | Insignificant change |  |
|  | **MTHFD1** | Methylenetetrahydrofolate dehydrogenase 1 | Important in amino acid and one-carbon metabolism | 39.836 vs. 44.937 | 0.887 | **1.31479999998518E-06** | Downregulated |  | 10.568 vs. 12.29 | 0.860 | 9.987000E-01 | Insignificant change |  |
| **Redox Balance & Detoxification** | **NRF2 (NFE2L2)** | Master regulator of oxidative stress response | Upregulated in chemo-resistant tumors | 71.949 vs. 111.517 | 0.645 | **1.62447832963153E-12** | Downregulated |  | 22.09 vs. 16.565 | 1.334 | **2.736400E-03** | Upregulated |  |
|  | **SOD2** | Superoxide dismutase | Neutralizes superoxide radicals | 210.03 vs. 455.919 | 0.461 | **1.62669877568078E-12** | Downregulated |  | 9.877 vs. 7.464 | 1.323 | 4.136000E-01 | Insignificant change |  |
|  | **GPX4** | Glutathione peroxidase | Regulates ferroptosis (iron-dependent cell death) | 371.489 vs. 415.664 | 0.894 | **1.480380E-03** | Downregulated |  | 234.498 vs. 177.773 | 1.319 | **3.255600E-02** | Upregulated |  |
|  | **GCLC** | Glutamate-cysteine ligase | Rate-limiting enzyme in glutathione synthesis | 17.233 vs. 24.648 | 0.699 | **8.59690000298841E-08** | Downregulated |  | 6.546 vs. 8.13 | 0.805 | 2.204600E-01 | Insignificant change |  |
| **Autophagy & ER Stress** | **LC3 (MAP1LC3B)** | Autophagy marker | Key for autophagosome formation | 60.803 vs. 92.549 | 0.657 | **1.62447832963153E-12** | Downregulated |  | 23.17 vs. 27.282 | 0.849 | 3.564200E-01 | Insignificant change |  |
|  | **ATG7** | Autophagy-related protein 7 | Essential for autophagy initiation | 13.206 vs. 12.388 | 1.066 | **3.11750000037136E-07** | Upregulated |  | 3.596 vs. 3.935 | 0.914 | 3.182600E-01 | Insignificant change |  |
|  | **CHOP (DDIT3)** | ER stress-induced apoptosis marker | Upregulated during severe ER stress | 29.991 vs. 33.07 | 0.908 | 7.457700E-02 | Insignificant change |  | 12.573 vs. 10.896 | 1.154 | 4.996800E-01 | Insignificant change |  |
|  | **BiP (GRP78, HSPA5)** | ER chaperone | Protects cancer cells from ER stress | 326.135 vs. 225.065 | 1.448 | **1.62447832963153E-12** | Upregulated |  | 149.079 vs. 126.539 | 1.178 | 7.152600E-01 | Insignificant change |  |

BRSA**:** breast invasive carcinoma

MET500: metastatic breast cancer

Yellow cells: gene expression significantly increased or decreased in both cancer subtypes
