# Supplementary material for: Mapping metabolic reprogramming in lung and breast cancer through integrative bioinformatics
Source: PLoS One. 2026 Jun 4;21(6):e0350628. doi: 10.1371/journal.pone.0350628 (PMC13235884; doi:10.1371/journal.pone.0350628)
Supplement: S2 Table — (DOCX) [file pone.0350628.s002.docx]

Supplementary Table S1. Full list of 289 analyzed genes with corresponding drug–gene interaction scores from DGIdb.

| **Gene** | **Drug** | **Regulatory approval** | **Indication** | **Interaction score** |
| --- | --- | --- | --- | --- |
| LDHA | CHEMBL:CHEMBL2058994 | Not Approved |  | 52.20379856 |
| COX4I1 | COMPOUND 9 [PMID: 11606127] | Not Approved |  | 52.20379856 |
| PFKFB3 | PFK-158 | Not Approved |  | 52.20379856 |
| ASNS | ASPARAGINASE | Approved |  | 11.60084412 |
| TK1 | PENCICLOVIR | Approved |  | 8.700633093 |
| TK1 | VALNIVUDINE HYDROCHLORIDE | Not Approved |  | 8.700633093 |
| TK1 | ARGININE BUTYRATE | Not Approved |  | 8.700633093 |
| PGD | PRALMORELIN | Not Approved |  | 8.700633093 |
| SCD | TIOCARLIDE | Not Approved |  | 7.457685508 |
| SCD | ARAMCHOL | Not Approved |  | 7.457685508 |
| DHODH | FARUDODSTAT | Not Approved |  | 5.49513669 |
| DHODH | BREQUINAR | Not Approved |  | 5.49513669 |
| DHODH | EMVODODSTAT | Not Approved |  | 5.49513669 |
| DHODH | TERIFLUNOMIDE | Approved | for treatment of multiple sclerosis | 5.49513669 |
| SLC2A1 | THIOCTIC ACID | Approved |  | 5.220379856 |
| PGD | PENICILLAMINE | Approved |  | 4.971790339 |
| ACADVL | XYT528B | Not Approved |  | 4.745799869 |
| ACADVL | COMPOUND 15 [PMID: 25411721] | Not Approved |  | 4.745799869 |
| ACADVL | COMPOUND 77A [PMID: 37639383] | Not Approved |  | 4.745799869 |
| ACADVL | COMPOUND 11D [PMID: 34406006] | Not Approved |  | 4.745799869 |
| ACADVL | BETULINIC ACID | Not Approved |  | 4.745799869 |
| LDHA | ANTHRACYCLINE ANTINEOPLASTIC ANTIBIOTIC | Not Approved |  | 4.350316547 |
| CPT1A | COMPOUND 10 [PMID: 31663733] | Not Approved |  | 4.015676812 |
| RRM2 | LY-2334737 | Not Approved |  | 4.015676812 |
| RRM2 | GEMCITABINE ELAIDATE | Not Approved |  | 4.015676812 |
| FASN | DENIFANSTAT | Not Approved |  | 3.866948041 |
| RRM2 | TRIAPINE | Not Approved | antineoplastic agent | 3.011757609 |
| G6PD | RASBURICASE | Approved |  | 2.923412719 |
| DHODH | S312 | Not Approved |  | 2.747568345 |
| DHODH | IFN-&BETA; | Not Approved |  | 2.747568345 |
| DHODH | S416 | Not Approved |  | 2.747568345 |
| DHODH | BRD9185 | Not Approved |  | 2.747568345 |
| DHODH | DSM705 | Not Approved |  | 2.747568345 |
| DHODH | ORLUDODSTAT | Not Approved |  | 2.747568345 |
| DHODH | ORLUDODSTAT | Not Approved |  | 2.747568345 |
| DHODH | COMPOUND 19 [PMID: 35925768] | Not Approved |  | 2.747568345 |
| DHODH | DSM421 | Not Approved |  | 2.747568345 |
| G6PD | PHENAZOPYRIDINE | Approved |  | 2.610189928 |
| SLC2A1 | BASIC FIBROBLAST GROWTH FACTOR | Not Approved |  | 2.610189928 |
| SLC2A1 | IL-7 | Not Approved |  | 2.610189928 |
| GCLC | BUTHIONINE SULFOXIMINE | Not Approved |  | 2.610189928 |
| SCD | MK-8245 | Not Approved |  | 2.485895169 |
| ACADVL | DEOXYCHOLIC ACID | Approved |  | 2.372899934 |
| ACADVL | INT-767 | Not Approved |  | 2.372899934 |
| TK1 | RILAPLADIB | Not Approved |  | 2.175158273 |
| TK1 | DEOXYCYTIDINE | Not Approved |  | 2.175158273 |
| G6PD | PEGLOTICASE | Approved |  | 2.088151942 |
| CPT1A | AZALANSTAT | Not Approved |  | 2.007838406 |
| RRM2 | TRANSFERRIN RECEPTOR-TARGETED ANTI-RRM2 SIRNA CALAA-01 | Not Approved |  | 2.007838406 |
| FASN | CERULENIN | Not Approved |  | 1.933474021 |
| FASN | LORGLUMIDE SODIUM | Not Approved |  | 1.933474021 |
| FASN | PRETOMANID | Approved |  | 1.933474021 |
| FASN | CHEMBL:CHEMBL578883 | Not Approved |  | 1.933474021 |
| FASN | GSK2194069 | Not Approved |  | 1.933474021 |
| FASN | CHEMBL:CHEMBL577862 | Not Approved |  | 1.933474021 |
| SOD2 | BILIRUBIN | Not Approved |  | 1.740126619 |
| GCLC | GSK852 | Not Approved |  | 1.740126619 |
| DHODH | VIDOFLUDIMUS | Not Approved | DMARD,antiinflammatory agent | 1.648541007 |
| G6PD | PAMAQUINE | Not Approved |  | 1.566113957 |
| G6PD | SULFANILAMIDE | Approved |  | 1.566113957 |
| PKM | PYRUVATE KINASE INHIBITOR TLN-232 | Not Approved |  | 1.450105516 |
| PKM | TT-232 | Not Approved | antineoplastic agent | 1.450105516 |
| PKM | CHEMBL:CHEMBL1091502 | Not Approved |  | 1.450105516 |
| PKM | CHEMBL:CHEMBL585265 | Not Approved |  | 1.450105516 |
| DHODH | VIDOFLUDIMUS | Not Approved |  | 1.373784173 |
| DHODH | ANIFROLUMAB | Not Approved |  | 1.373784173 |
| GCLC | XD14 | Not Approved |  | 1.305094964 |
| CPT1A | PERHEXILINE | Not Approved | Antianginal Agents; Vasodilator Agents | 1.147336232 |
| RRM2 | MOTEXAFIN GADOLINIUM | Not Approved | antineoplastic agent | 1.147336232 |
| DHODH | LEFLUNOMIDE | Approved | Antirheumatic Agents | 1.144820144 |
| G6PD | CO-TRIMOXAZOLE | Not Approved |  | 1.044075971 |
| G6PD | SODIUM ASCORBATE | Approved |  | 1.044075971 |
| G6PD | SITAMAQUINE | Not Approved |  | 1.044075971 |
| SLC2A1 | THYMIDINE | Approved |  | 1.044075971 |
| GCLC | (+)-JQ1 | Not Approved |  | 1.044075971 |
| GCLC | WNY0824 | Not Approved |  | 1.044075971 |
| MTHFD1 | PEMETREXED DISODIUM | Approved | Antineoplastic Agents,antineoplastic agent | 1.003919203 |
| RRM2 | TEZACITABINE | Not Approved |  | 1.003919203 |
| ACADVL | OLEANOLIC ACID | Not Approved |  | 0.949159974 |
| SCD | CLOFIBRATE | Approved | Anticholesteremic Agents | 0.932210689 |
| PGD | PHENOBARBITAL | Approved | Anticonvulsants; Hypnotics and Sedatives | 0.915856115 |
| G6PD | TAFENOQUINE | Approved |  | 0.783056978 |
| GCLC | SULFAMETHOXAZOLE | Approved |  | 0.745768551 |
| PKM | CHEMBL:CHEMBL1093369 | Not Approved |  | 0.725052758 |
| SOD2 | ASPARAGINASE | Approved |  | 0.725052758 |
| SCD | ROSIGLITAZONE | Approved | for treatment of Alzheimer's disease,antidiabetic | 0.67797141 |
| TK1 | ZIDOVUDINE | Approved |  | 0.64449134 |
| FASN | CHEMBL:CHEMBL1255647 | Not Approved |  | 0.64449134 |
| FASN | GT 389-255 | Not Approved | antiobesity agent | 0.64449134 |
| SOD2 | ANTICONVULSANT AGENT | Not Approved |  | 0.580042206 |
| RRM2 | GALLIUM NITRATE | Approved |  | 0.573668116 |
| SCD | VITAMIN A | Approved |  | 0.552421149 |
| CPT1A | PEGASPARGASE | Approved |  | 0.535423575 |
| RRM2 | CLADRIBINE | Approved |  | 0.535423575 |
| G6PD | SODIUM SULFATE ANHYDROUS | Approved |  | 0.522037986 |
| G6PD | SULFADIAZINE | Approved |  | 0.522037986 |
| G6PD | NALIDIXIC ACID | Approved |  | 0.522037986 |
| G6PD | SULFACETAMIDE | Approved |  | 0.522037986 |
| G6PD | DRUGSATFDA.NDA:021717 | Not Approved |  | 0.522037986 |
| G6PD | METABUTETHAMINE | Not Approved |  | 0.522037986 |
| G6PD | CARBASALATE CALCIUM | Not Approved |  | 0.522037986 |
| G6PD | SODIUM NITRATE | Approved |  | 0.522037986 |
| G6PD | ARTICAINE | Approved |  | 0.522037986 |
| SOD2 | ANTIBIOTIC | Not Approved |  | 0.511801947 |
| CPT1A | LEVOCARNITINE PROPIONATE | Not Approved | for treatment of intermittent claudication | 0.501959602 |
| GCLC | ANTIOXIDANT | Not Approved |  | 0.497179034 |
| FASN | CHEMBL:CHEMBL585030 | Not Approved |  | 0.483368505 |
| FASN | CHEMBL:CHEMBL604664 | Not Approved |  | 0.483368505 |
| SLC2A1 | RECOMBINANT FIBROBLAST GROWTH FACTOR 2 | Not Approved |  | 0.474579987 |
| ACADVL | CHENODEOXYCHOLIC ACID | Approved |  | 0.474579987 |
| SOD2 | ANTIOXIDANT | Not Approved |  | 0.414315862 |
| ACADVL | CHOLIC ACID | Approved |  | 0.395483322 |
| G6PD | MAFENIDE | Approved |  | 0.391528489 |
| FASN | DNQX | Not Approved |  | 0.386694804 |
| RRM2 | HYDROXYUREA | Approved | Antineoplastic Agents | 0.382445411 |
| ACADVL | LITHOCHOLIC ACID | Not Approved |  | 0.365061528 |
| PKM | CHEMBL:CHEMBL1214407 | Not Approved |  | 0.362526379 |
| G6PD | SODIUM NITRITE | Approved |  | 0.348025324 |
| NDUFS3 | NV-128 | Not Approved |  | 0.348025324 |
| NDUFS3 | ME-344 | Not Approved |  | 0.341201298 |
| SLC2A1 | GLUFOSFAMIDE | Not Approved | antineoplastic agent | 0.326273741 |
| NDUFS3 | METFORMIN HYDROCHLORIDE | Approved |  | 0.310736896 |
| SLC2A1 | SELENIUM | Approved |  | 0.307081168 |
| SLC2A1 | PIOGLITAZONE HYDROCHLORIDE | Approved | antidiabetic | 0.307081168 |
| DHODH | IMMUNOSUPPRESSANT | Not Approved |  | 0.305285372 |
| FASN | ORLISTAT | Approved | antiobesity agent | 0.297457542 |
| PKM | CHEMBL:CHEMBL261693 | Not Approved |  | 0.290021103 |
| PKM | CHEMBL:CHEMBL429335 | Not Approved |  | 0.290021103 |
| PKM | 3-HYDROXYBENZYLHYDRAZINE | Not Approved |  | 0.290021103 |
| SCD | COLCHICINE | Approved | for treatment of gout | 0.286834058 |
| G6PD | NITROFURANTOIN, MACROCRYSTALS | Approved |  | 0.281097377 |
| FASN | CHEMBL:CHEMBL586937 | Not Approved |  | 0.276210574 |
| FASN | ANTINEOPLASTIC AGENT | Not Approved |  | 0.276210574 |
| MTHFD1 | CISPLATIN | Approved |  | 0.266345911 |
| G6PD | AMODIAQUINE | Not Approved |  | 0.261018993 |
| G6PD | SULFISOXAZOLE | Approved |  | 0.261018993 |
| G6PD | ACETAMINOPHEN / CODEINE | Not Approved |  | 0.261018993 |
| G6PD | PENICILLIN | Not Approved |  | 0.261018993 |
| G6PD | EPIANDROSTERONE | Not Approved |  | 0.261018993 |
| G6PD | POTASSIUM CHLORIDE | Approved |  | 0.261018993 |
| G6PD | TRIAPINE | Not Approved | antineoplastic agent | 0.261018993 |
| G6PD | ARTICAINE / EPINEPHRINE | Not Approved |  | 0.261018993 |
| SLC2A1 | PLATINUM | Not Approved |  | 0.261018993 |
| CPT1A | THIOGUANINE | Approved |  | 0.243374352 |
| SLC2A1 | ROSIGLITAZONE | Approved | for treatment of Alzheimer's disease,antidiabetic | 0.237289993 |
| FASN | SELENIUM | Approved |  | 0.227467532 |
| SLC2A1 | GENTAMICIN | Approved |  | 0.226973037 |
| SLC2A1 | TRIAMCINOLONE | Approved | for treatment of diabetic macular edema | 0.226973037 |
| RRM2 | CLOFARABINE | Approved | antineoplastic agent | 0.211351411 |
| G6PD | ROPIVACAINE | Approved | anestethic | 0.208815194 |
| G6PD | ZINC CHLORIDE | Approved |  | 0.208815194 |
| G6PD | DIMERCAPROL | Approved |  | 0.208815194 |
| SLC2A1 | TCDD | Not Approved |  | 0.208815194 |
| PKM | CIS-RESVERATROL | Not Approved |  | 0.207157931 |
| PKM | CHEMBL:CHEMBL1333049 | Not Approved |  | 0.207157931 |
| PKM | CHEMBL:CHEMBL1303948 | Not Approved |  | 0.207157931 |
| FASN | ADRIAMYCIN | Not Approved |  | 0.203523581 |
| G6PD | SULFAMETHOXAZOLE | Approved |  | 0.186442138 |
| PKM | SILVER SULFADIAZINE | Approved |  | 0.181263189 |
| PKM | CHEMBL:CHEMBL473721 | Not Approved |  | 0.181263189 |
| SLC2A1 | DIAZEPAM | Approved | anticonvulsant | 0.180013098 |
| CPT1A | MERCAPTOPURINE | Approved | Antineoplastic Agents | 0.178474525 |
| MTHFD1 | DOXORUBICIN HYDROCHLORIDE | Approved | antineoplastic agent | 0.176364184 |
| G6PD | FURAZOLIDONE | Approved |  | 0.174012662 |
| G6PD | SULFAMETHAZINE | Approved |  | 0.174012662 |
| G6PD | NITROFURAZONE | Approved |  | 0.174012662 |
| G6PD | NORETHINDRONE ACETATE | Approved |  | 0.174012662 |
| PKM | CHEMBL:CHEMBL125044 | Not Approved |  | 0.161122835 |
| SOD2 | CYCLOPHOSPHAMIDE ANHYDROUS | Approved |  | 0.161122835 |
| SLC2A1 | THERAPEUTIC GLUCOCORTICOID | Not Approved |  | 0.153540584 |
| SOD2 | VALPROIC ACID | Approved | Anticonvulsants,for treatment of basal cell carcinoma,anticonvulsant | 0.152642686 |
| G6PD | TOLAZAMIDE | Approved | Hypoglycemic Agents | 0.14915371 |
| G6PD | SUCCIMER | Approved |  | 0.14915371 |
| G6PD | GLIPIZIDE | Approved | Hypoglycemic Agents | 0.14915371 |
| FASN | PENTABROMOPHENOL | Not Approved |  | 0.148728771 |
| PKM | CHEMBL:CHEMBL595227 | Not Approved |  | 0.145010552 |
| MTHFD1 | GEMCITABINE | Approved | antineoplastic agent | 0.141858148 |
| GCLC | DEHYDRATED ALCOHOL | Approved |  | 0.132161515 |
| PKM | CHEMBL:CHEMBL408850 | Not Approved |  | 0.131827774 |
| PKM | TRANILAST | Not Approved |  | 0.131827774 |
| PKM | CHEMBL:CHEMBL154580 | Not Approved |  | 0.131827774 |
| RRM2 | GEMCITABINE | Approved | antineoplastic agent | 0.130945983 |
| G6PD | MOXIFLOXACIN | Approved |  | 0.130509496 |
| G6PD | NORFLOXACIN | Approved |  | 0.130509496 |
| G6PD | ACETAMINOPHEN/TRAMADOL | Not Approved |  | 0.130509496 |
| G6PD | GLIMEPIRIDE | Approved | antidiabetic | 0.130509496 |
| G6PD | TOLBUTAMIDE | Approved | Hypoglycemic Agents | 0.130509496 |
| SLC2A1 | PHENYTOIN SODIUM | Approved | Anticonvulsants | 0.124294758 |
| GCLC | CARBOPLATIN | Approved |  | 0.121404183 |
| PKM | NITAZOXANIDE | Approved |  | 0.120842126 |
| G6PD | QUININE | Approved |  | 0.120470304 |
| RRM2 | FLUDARABINE | Approved | antineoplastic agent | 0.118108142 |
| FASN | LIOTHYRONINE | Approved | Hormone Replacement Agents | 0.117180244 |
| G6PD | PTHRP | Not Approved |  | 0.116008441 |
| G6PD | ATAZANAVIR | Approved |  | 0.116008441 |
| FASN | EPIGALLOCATECHIN GALLATE | Approved |  | 0.113733766 |
| FASN | CHEMBL:CHEMBL523200 | Not Approved |  | 0.113733766 |
| PKM | AMLEXANOX | Approved |  | 0.111546578 |
| SLC2A1 | ESTRADIOL VALERATE | Approved | for treatment of menopausal symptoms,contraceptive,treatment for menopause,hormone replacement | 0.111071912 |
| G6PD | GLYBURIDE | Approved | Hypoglycemic Agents,antidiabetic | 0.108007859 |
| SOD2 | DOCETAXEL ANHYDROUS | Approved | antineoplastic agent | 0.104826905 |
| G6PD | GLICLAZIDE | Approved | Hypoglycemic Agents | 0.104407597 |
| G6PD | NICORANDIL | Not Approved |  | 0.104407597 |
| PKM | CHEMBL:CHEMBL599013 | Not Approved |  | 0.103578965 |
| PKM | ERBSTATIN | Not Approved |  | 0.103578965 |
| PKM | SB 206553 | Not Approved |  | 0.103578965 |
| PKM | CHEMBL:CHEMBL584458 | Not Approved |  | 0.103578965 |
| FASN | FENTICLOR | Not Approved |  | 0.101761791 |
| CPT1A | CYTARABINE | Approved | antineoplastic agent | 0.10039192 |
| RRM2 | CYTARABINE | Approved | antineoplastic agent | 0.10039192 |
| CPT1A | CYCLOPHOSPHAMIDE ANHYDROUS | Approved |  | 0.099152514 |
| CPT1A | VINCRISTINE | Approved | antineoplastic agent | 0.099152514 |
| SOD2 | DAUNORUBICIN LIPOSOMAL | Approved | antineoplastic agent | 0.095611353 |
| G6PD | PROGESTIN | Approved | contraceptive | 0.094915997 |
| G6PD | CEFTRIAXONE | Approved |  | 0.094915997 |
| G6PD | PRILOCAINE | Approved | for treatment of premature ejaculation,anesthetic | 0.094915997 |
| G6PD | CARMUSTINE | Approved | antineoplastic agent | 0.094915997 |
| G6PD | CHLORPROPAMIDE | Approved | Hypoglycemic Agents | 0.094915997 |
| G6PD | CIPROFLOXACIN | Approved |  | 0.094915997 |
| SLC2A1 | GENISTEIN | Approved |  | 0.090006549 |
| SLC2A1 | PROGESTERONE | Approved | for reducing the risk of pre-term birth for women with short cervix a mid-pregnancy,for prevention of preterm delivery,for symptomatic treatment of menopausal symptoms,neuroprotectant for stroke victims | 0.088481015 |
| CPT1A | DAUNORUBICIN LIPOSOMAL | Approved | antineoplastic agent | 0.088256633 |
| G6PD | BROXURIDINE | Not Approved |  | 0.087006331 |
| G6PD | PERINDOPRIL | Approved | Antihypertensive Agents | 0.087006331 |
| DHODH | PEGINTERFERON ALFA-2B | Approved |  | 0.085861511 |
| PKM | PSEUDOEPHEDRINE | Approved | antiallergy agent | 0.085300324 |
| G6PD | ASCORBIC ACID | Approved |  | 0.080313536 |
| G6PD | CHLOROPROCAINE | Approved | Local,Anesthetics | 0.080313536 |
| SOD2 | METHOTREXATE | Approved | DMARD | 0.079096664 |
| SOD2 | PACLITAXEL | Approved | for treatment of peripheral arterial disease (PAD),DMARD,antiinflammatory agent,antineoplastic agent | 0.079096664 |
| FASN | PYROGALLOL RED | Not Approved |  | 0.077338961 |
| PKM | INDIRUBIN-3'-MONOXIME | Not Approved |  | 0.076321343 |
| PKM | TYRPHOSTIN B48 | Not Approved |  | 0.076321343 |
| G6PD | NIMESULIDE | Not Approved |  | 0.074576855 |
| G6PD | METOCLOPRAMIDE HYDROCHLORIDE | Approved | antimigraine agent,antiemetic,for treatment of gastroesophageal reflux disease,motilitant,for treatment of diabetic gastroparesis | 0.074576855 |
| CPT1A | METHOTREXATE | Approved | DMARD | 0.073012306 |
| PKM | HYDRALAZINE | Approved | antihypertensive agent | 0.072505276 |
| GCLC | CISPLATIN | Approved |  | 0.071025576 |
| G6PD | MELOXICAM | Approved | NSAID | 0.065254748 |
| G6PD | SULFASALAZINE | Approved | DMARD,antiinflammatory agent | 0.065254748 |
| G6PD | MEPIVACAINE HYDROCHLORIDE | Approved | anestethic | 0.065254748 |
| PKM | THIRAM | Not Approved |  | 0.063048066 |
| PKM | DECYNIUM 22 | Not Approved |  | 0.063048066 |
| SOD2 | DOXORUBICIN HYDROCHLORIDE | Approved | antineoplastic agent | 0.058788061 |
| G6PD | DABRAFENIB | Approved | antineoplastic agent | 0.058004221 |
| G6PD | VITAMIN K | Approved | Antifibrinolytic Agents | 0.058004221 |
| PKM | SANGUINARIUM | Not Approved |  | 0.058004221 |
| FASN | RISPERIDONE | Approved | Antipsychotic Agents,antipsychotic agent | 0.057715642 |
| SLC2A1 | TRETINOIN | Approved | for treatment of acne | 0.057366812 |
| G6PD | BUSULFAN | Approved |  | 0.054951367 |
| CPT1A | DOXORUBICIN HYDROCHLORIDE | Approved | antineoplastic agent | 0.054265903 |
| PKM | NICLOSAMIDE | Approved |  | 0.053707612 |
| G6PD | FLUTAMIDE | Approved | Hormonal,Antineoplastic Agents | 0.052203799 |
| G6PD | QUINACRINE | Not Approved |  | 0.052203799 |
| PKM | 4-CHLOROMERCURIBENZOIC ACID | Not Approved |  | 0.051789483 |
| G6PD | PROBENECID | Approved | Uricosuric Agents | 0.047457999 |
| G6PD | TETRACAINE | Approved |  | 0.047457999 |
| G6PD | PRIMAQUINE | Approved |  | 0.047457999 |
| G6PD | DAPSONE | Approved |  | 0.047457999 |
| FASN | HEXACHLOROPHENE | Approved |  | 0.047157903 |
| G6PD | TRIAMCINOLONE | Approved | for treatment of diabetic macular edema | 0.045394607 |
| G6PD | OFLOXACIN | Approved |  | 0.043503165 |
| G6PD | PROPRANOLOL HYDROCHLORIDE | Approved | for treatment of cancer cachexia | 0.043503165 |
| G6PD | PROTOPORPHYRIN | Not Approved |  | 0.041763039 |
| PKM | EBSELEN | Not Approved |  | 0.041431586 |
| G6PD | NITROGLYCERIN | Approved | for treatment of Raynaud's disease | 0.040156768 |
| FASN | CLOZAPINE | Approved | Antipsychotic Agents | 0.039865444 |
| PKM | PD-98059 | Not Approved |  | 0.039192041 |
| G6PD | PREDNISOLONE | Approved | corticosteroid,antiinflammatory agent | 0.037288428 |
| G6PD | DIAZEPAM | Approved | anticonvulsant | 0.03600262 |
| FASN | PACLITAXEL | Approved | for treatment of peripheral arterial disease (PAD),DMARD,antiinflammatory agent,antineoplastic agent | 0.035154073 |
| G6PD | CHLOROQUINE | Approved |  | 0.032627374 |
| G6PD | LIDOCAINE | Approved | for treatment of pelvic pain of bladder origin and interstitital cystitis,for treatment of premature ejaculation,anestethic,anesthetic | 0.030708117 |
| G6PD | BUPIVACAINE | Approved | neuralgia,analgesic,local anestethic,Local,Anesthetics | 0.030708117 |
| G6PD | PIOGLITAZONE HYDROCHLORIDE | Approved | antidiabetic | 0.030708117 |
| G6PD | HYDROXYCHLOROQUINE | Approved | antirheumatic agent | 0.02900211 |
| G6PD | PRASTERONE | Approved | hormone supplement for increasing bone mineral density in patients with systemic lupus erythematosus | 0.02900211 |
| G6PD | OMEPRAZOLE | Approved | antiulcer agent,Proton pump inhibitor | 0.028218269 |
| G6PD | OXYMETAZOLINE HYDROCHLORIDE | Approved | analgesic | 0.026101899 |
| G6PD | THERAPEUTIC CORTICOSTEROID | Not Approved |  | 0.024858952 |
| G6PD | MESALAMINE | Approved | for treatment of ulcerative proctitis,for treatment of ulcerative colitis,antiinflammatory agent | 0.023728999 |
| G6PD | ERYTHROMYCIN | Approved |  | 0.022697304 |
| G6PD | TRAMETINIB DIMETHYL SULFOXIDE | Approved | antineoplastic agent | 0.022214382 |
| G6PD | SODIUM CHLORIDE | Approved |  | 0.018644214 |
| G6PD | EBSELEN | Not Approved |  | 0.014915371 |
| G6PD | METHYLENE BLUE ANHYDROUS | Approved |  | 0.014109135 |
| G6PD | EPINEPHRINE | Approved | anestethic | 0.01305095 |
| G6PD | CISPLATIN | Approved |  | 0.007102558 |
| G6PD | DOXORUBICIN HYDROCHLORIDE | Approved | antineoplastic agent | 0.007054567 |
