# Supplementary material for: Mapping metabolic reprogramming in lung and breast cancer through integrative bioinformatics
Source: PLoS One. 2026 Jun 4;21(6):e0350628. doi: 10.1371/journal.pone.0350628 (PMC13235884; doi:10.1371/journal.pone.0350628)
Supplement: S3 Table — (DOCX) [file pone.0350628.s003.docx]

Supplementary Table S2. Full List of KEGG Pathways Enriched from the Selected Gene Set Using Enrichr

| **Pathway** | **Overlap** | **P-value** | **Adjusted  P-value** | **Odds Ratio** | **Combined  Score** | **Genes** |
| --- | --- | --- | --- | --- | --- | --- |
| Pentose phosphate pathway | 10990 | 0.0000455 | 0.00082 | 285.21 | 2851.75 | G6PD;PGD |
| Pyrimidine metabolism | 20486 | 0.0002 | 0.00100 | 147.70 | 1290.68 | TK1;DHODH |
| Glutathione metabolism | 20852 | 0.0002 | 0.00100 | 145.00 | 1262.00 | G6PD;PGD |
| Central carbon metabolism in cancer | 25600 | 0.0003 | 0.00113 | 117.21 | 971.77 | G6PD;SLC2A1 |
| Diabetic cardiomyopathy | 2/203 | 0.0021 | 0.00749 | 39.39 | 243.20 | G6PD;SLC2A1 |
| Alanine, aspartate and glutamate metabolism | 13516 | 0.0129 | 0.03846 | 92.39 | 402.10 | ASNS |
| Fatty acid degradation | 15707 | 0.0150 | 0.03846 | 79.17 | 332.73 | ACADVL |
| Adipocytokine signaling pathway | 25204 | 0.0239 | 0.04504 | 48.84 | 182.34 | SLC2A1 |
| Renal cell carcinoma | 25204 | 0.0239 | 0.04504 | 48.84 | 182.34 | SLC2A1 |
| Insulin secretion | 31413 | 0.0297 | 0.04504 | 39.04 | 137.25 | SLC2A1 |
| Bile secretion | 32874 | 0.0311 | 0.04504 | 37.27 | 129.38 | SLC2A1 |
| Glucagon signaling pathway | 1/107 | 0.0369 | 0.04504 | 31.27 | 103.21 | SLC2A1 |
| Drug metabolism | 1/108 | 0.0372 | 0.04504 | 30.98 | 101.95 | TK1 |
| Insulin resistance | 1/108 | 0.0372 | 0.04504 | 30.98 | 101.95 | SLC2A1 |
| HIF-1 signaling pathway | 1/109 | 0.0375 | 0.04504 | 30.69 | 100.73 | SLC2A1 |
| Thyroid hormone signaling pathway | 1/121 | 0.0416 | 0.04679 | 27.60 | 87.77 | SLC2A1 |
| Human T-cell leukemia virus 1 infection | 1/219 | 0.0742 | 0.07855 | 15.12 | 39.33 | SLC2A1 |
| Pathways in cancer | 1/531 | 0.1717 | 0.17171 | 6.12 | 10.78 | SLC2A1 |
