# Supplementary material for: Mapping metabolic reprogramming in lung and breast cancer through integrative bioinformatics
Source: PLoS One. 2026 Jun 4;21(6):e0350628. doi: 10.1371/journal.pone.0350628 (PMC13235884; doi:10.1371/journal.pone.0350628)
Supplement: S4 Table — (DOCX) [file pone.0350628.s004.docx]

Supplementary Table S3. Full List of GO Biological Processes Enriched from the Selected Gene Set Using Enrichr

| **Biological Process** | **Overlap** | **P-value** | **Adjusted P-value** | **Odds Ratio** | **Combined Score** | **Genes** |
| --- | --- | --- | --- | --- | --- | --- |
| L-ascorbic Acid Metabolic Process (GO:0019852) | 45778 | 0.001749 | 0.01929 | 832.88 | 5287.72 | SLC2A1 |
| Pyrimidine Ribonucleotide Metabolic Process (GO:0009218) | 45778 | 0.001749 | 0.01929 | 832.88 | 5287.72 | DHODH |
| Monosaccharide Metabolic Process (GO:0005996) | 45839 | 0.002448 | 0.01929 | 555.19 | 3338.15 | SLC2A1 |
| Pyrimidine Ribonucleotide Biosynthetic Process (GO:0009220) | 45839 | 0.002448 | 0.01929 | 555.19 | 3338.15 | DHODH |
| Negative Regulation of Fatty Acid Oxidation (GO:0046322) | 45839 | 0.002448 | 0.01929 | 555.19 | 3338.15 | ACADVL |
| Long-Chain Fatty Acid Import Into Cell (GO:0044539) | 45931 | 0.003495 | 0.01929 | 370.07 | 2093.27 | SLC2A1 |
| Mitotic DNA Replication (GO:1902969) | 45931 | 0.003495 | 0.01929 | 370.07 | 2093.27 | TK1 |
| Fatty Acid Beta-Oxidation Using acyl-CoA Dehydrogenase (GO:0033539) | 45931 | 0.003495 | 0.01929 | 370.07 | 2093.27 | ACADVL |
| Fatty Acid Transmembrane Transport (GO:1902001) | 45962 | 0.003844 | 0.01929 | 333.05 | 1852.16 | SLC2A1 |
| Dehydroascorbic Acid Transport (GO:0070837) | 45992 | 0.004193 | 0.01929 | 302.76 | 1657.40 | SLC2A1 |
| Negative Regulation of Fatty Acid Biosynthetic Process (GO:0045717) | 41640 | 0.004890 | 0.01977 | 256.15 | 1362.86 | ACADVL |
| Regulation of Fatty Acid Oxidation (GO:0046320) | 42005 | 0.005239 | 0.01977 | 237.85 | 1249.08 | ACADVL |
| D-glucose Import (GO:0046323) | 42370 | 0.005587 | 0.01977 | 221.98 | 1151.45 | SLC2A1 |
| Regulation of Steroid Metabolic Process (GO:0019218) | 44562 | 0.007676 | 0.02085 | 158.51 | 771.89 | ACADVL |
| Hexose Transmembrane Transport (GO:0008645) | 44562 | 0.007676 | 0.02085 | 158.51 | 771.89 | SLC2A1 |
| D-glucose Transmembrane Transport (GO:1904659) | 44562 | 0.007676 | 0.02085 | 158.51 | 771.89 | SLC2A1 |
| Regulation of Cholesterol Metabolic Process (GO:0090181) | 45292 | 0.008371 | 0.02085 | 144.71 | 692.15 | ACADVL |
| Negative Regulation of Lipid Biosynthetic Process (GO:0051055) | 46023 | 0.009066 | 0.02085 | 133.12 | 626.10 | ACADVL |
| Regulation of Fatty Acid Biosynthetic Process (GO:0042304) | 46023 | 0.009066 | 0.02085 | 133.12 | 626.10 | ACADVL |
| Negative Regulation of Fatty Acid Metabolic Process (GO:0045922) | 46023 | 0.009066 | 0.02085 | 133.12 | 626.10 | ACADVL |
| Positive Regulation of Mitotic Cell Cycle (GO:0045931) | 46753 | 0.009760 | 0.02138 | 123.25 | 570.56 | ASNS |
| Regulation of Small Molecule Metabolic Process (GO:0062012) | 12055 | 0.011495 | 0.02403 | 103.96 | 464.29 | ACADVL |
| Carboxylic Acid Metabolic Process (GO:0019752) | 13150 | 0.012534 | 0.02469 | 95.04 | 416.20 | SLC2A1 |
| Nucleobase-Containing Compound Metabolic Process (GO:0006139) | 13516 | 0.012880 | 0.02469 | 92.39 | 402.10 | TK1 |
| Vitamin Transport (GO:0051180) | 14977 | 0.014264 | 0.02625 | 83.14 | 353.34 | SLC2A1 |
| Water-Soluble Vitamin Metabolic Process (GO:0006767) | 16072 | 0.015301 | 0.02707 | 77.33 | 323.21 | SLC2A1 |
| Fatty Acid Beta-Oxidation (GO:0006635) | 17533 | 0.016682 | 0.02842 | 70.73 | 289.53 | ACADVL |
| Response to Peptide Hormone (GO:0043434) | 20455 | 0.019439 | 0.03192 | 60.42 | 238.08 | SLC2A1 |
| Protein Homotetramerization (GO:0051289) | 21551 | 0.020471 | 0.03192 | 57.28 | 222.76 | TK1 |
| Positive Regulation of Cell Cycle (GO:0045787) | 21916 | 0.020815 | 0.03192 | 56.31 | 218.04 | ASNS |
| Response to Insulin (GO:0032868) | 29952 | 0.028353 | 0.04021 | 40.97 | 145.98 | SLC2A1 |
| Protein Tetramerization (GO:0051262) | 30317 | 0.028695 | 0.04021 | 40.47 | 143.71 | TK1 |
| Vascular Transport (GO:0010232) | 30682 | 0.029036 | 0.04021 | 39.98 | 141.50 | SLC2A1 |
| Transport Across Blood-Brain Barrier (GO:0150104) | 31413 | 0.029719 | 0.04021 | 39.04 | 137.25 | SLC2A1 |
| Organic Anion Transport (GO:0015711) | 1/105 | 0.036181 | 0.04755 | 31.87 | 105.79 | SLC2A1 |
| Protein-Containing Complex Organization (GO:0043933) | 1/123 | 0.042270 | 0.05297 | 27.15 | 85.88 | SLC2A1 |
| Regulation of Mitotic Cell Cycle (GO:0007346) | 1/124 | 0.042607 | 0.05297 | 26.92 | 84.97 | ASNS |
| Cellular Response to Starvation (GO:0009267) | 1/141 | 0.048325 | 0.05779 | 23.63 | 71.61 | ASNS |
| Protein Homooligomerization (GO:0051260) | 1/143 | 0.048996 | 0.05779 | 23.30 | 70.27 | TK1 |
| Cellular Component Assembly (GO:0022607) | 1/277 | 0.093027 | 0.10698 | 11.91 | 28.28 | SLC2A1 |
| Central Nervous System Development (GO:0007417) | 1/288 | 0.096562 | 0.10834 | 11.44 | 26.75 | SLC2A1 |
| Protein-Containing Complex Assembly (GO:0065003) | 1/323 | 0.107733 | 0.11799 | 10.18 | 22.69 | SLC2A1 |
| Negative Regulation of Programmed Cell Death (GO:0043069) | 1/382 | 0.126296 | 0.13511 | 8.58 | 17.75 | ASNS |
| Negative Regulation of Apoptotic Process (GO:0043066) | 1/475 | 0.154884 | 0.15987 | 6.86 | 12.80 | ASNS |
| Nervous System Development (GO:0007399) | 1/480 | 0.156398 | 0.15987 | 6.79 | 12.60 | SLC2A1 |
| Regulation of Apoptotic Process (GO:0042981) | 1/704 | 0.221883 | 0.22188 | 4.57 | 6.89 | ASNS |
