# Supplementary material for: Mapping metabolic reprogramming in lung and breast cancer through integrative bioinformatics
Source: PLoS One. 2026 Jun 4;21(6):e0350628. doi: 10.1371/journal.pone.0350628 (PMC13235884; doi:10.1371/journal.pone.0350628)
Supplement: S5 Table — (DOCX) [file pone.0350628.s005.docx]

Supplementary Table S4. Full STRING Protein–Protein Interaction Data for the Selected Gene Set

| **#node1** | **node2** | **node1_string_id** | **node2_string_id** | **neighborhood_on_chromosome** | **gene_fusion** | **phylogenetic_cooccurrence** | **homology** | **coexpression** | **experimentally_determined_interaction** | **database_annotated** | **automated_textmining** | **combined_score** |
| --- | --- | --- | --- | --- | --- | --- | --- | --- | --- | --- | --- | --- |
| ASNS | SLC2A1 | 9606.ENSP00000377845 | 9606.ENSP00000416293 | 0 | 0 | 0 | 0 | 0.063 | 0 | 0 | 0.517 | 0.528 |
| G6PD | PGD | 9606.ENSP00000377192 | 9606.ENSP00000270776 | 0.111 | 0.899 | 0.457 | 0 | 0.666 | 0.225 | 0.585 | 0.956 | 0.999 |
| G6PD | SLC2A1 | 9606.ENSP00000377192 | 9606.ENSP00000416293 | 0 | 0 | 0 | 0 | 0.088 | 0 | 0 | 0.592 | 0.612 |
| PGD | G6PD | 9606.ENSP00000270776 | 9606.ENSP00000377192 | 0.111 | 0.899 | 0.457 | 0 | 0.666 | 0.225 | 0.585 | 0.956 | 0.999 |
| SLC2A1 | G6PD | 9606.ENSP00000416293 | 9606.ENSP00000377192 | 0 | 0 | 0 | 0 | 0.088 | 0 | 0 | 0.592 | 0.612 |
| SLC2A1 | ASNS | 9606.ENSP00000416293 | 9606.ENSP00000377845 | 0 | 0 | 0 | 0 | 0.063 | 0 | 0 | 0.517 | 0.528 |
| SLC2A1 | TK1 | 9606.ENSP00000416293 | 9606.ENSP00000468425 | 0 | 0 | 0 | 0 | 0.089 | 0 | 0.285 | 0.264 | 0.479 |
| TK1 | SLC2A1 | 9606.ENSP00000468425 | 9606.ENSP00000416293 | 0 | 0 | 0 | 0 | 0.089 | 0 | 0.285 | 0.264 | 0.479 |
